# Conceptual design of airborne contra rotating VAWTs for rooftop wind energy

**DOI:** 10.1038/s41598-025-90601-3

**Published:** 2025-02-26

**Authors:** Jayakrishnan Radhakrishnan, Surya Sridhar, Mohammed Zuber, Eddie Y. K. Ng, B. Satish Shenoy

**Affiliations:** 1 Department of Aeronautical and Automobile Engineering, Manipal Institute of Technology, Manipal Academy of Higher Education, Manipal, 576104 India; 2 Department of Aerospace Science and Technology, Politecnico di Milano, 20156 Milan, Italy; 3 School of Mechanical and Aerospace Engineering, Nanyang Technical University, NTU, Singapore, 639798 Singapore

**Keywords:** Wind energy, Engineering

## Abstract

Co-rotating, counter, and contra-rotating Vertical Axis Wind Turbines (VAWTs) offer higher power yields than singular turbines due to synergetic interactions, making them ideal for rooftop applications. This study focuses on enhancing the efficiency of a Contra-Rotating VAWT (CR-VAWT) using a ducted airborne configuration. A wind gathering device (WGD), optimized via the Taguchi method, is integrated around the CR-VAWT, which is elevated using an oblate spheroid aerostat designed through a force-weight approach. CFD analyses reveal that incorporating the WGD boosts power output by 32% and increases instantaneous torque by 40% during dynamic stall. The WGD enhances flow redirection towards the rotor plane, achieving a 58% power gain at a 10°skew angle. By suppressing blade-tip vortex shedding, the WGD not only improves efficiency but may also reduce noise. This portable system is suitable for rooftops with limited space, requiring fewer turbines to meet energy demands while reducing noise and improving aesthetics. It also allows for integration with other renewable technologies like photovoltaics, promoting sustainability and lowering carbon footprints. A comprehensive implementation framework is presented to support future research on airborne VAWTs.

## Introduction

With the rise in importance towards renewable energy and its generation in an off-grid urban setup to reduce greenhouse emissions, solar and wind power have been the forerunners for urban power production. The sophistication of an urban setup and the limited wind resources give rise to various problems in harnessing power from wind. Research on small-scale Horizontal and Vertical Axis Wind Turbines (HAWTs & VAWTs) has been gaining importance in the present day and age. Amongst the two, VAWTs are proven to be a more feasible and viable option for an urban setup due to their properties of omindirectionality towards incoming wind, ability to function efficiently under turbulent gusts^1–4^ and the reduced costs concerning the manufacturing and the maintenance of the same, out of which the former two properties are associated with the wind resources in urban setups^5–7^. For this reason, research on VAWTs has garnered more attention in recent years. Specifically, straight-bladed H-VAWTs have been the go-to choice due to the simplicity of their design and the overall efficiency & effectiveness compared to other types of VAWTs. However, the complex flow dynamics of cross-flow devices like VAWTs are one of the key reasons behind their reduced efficiency compared to their axial counterparts like HAWTs. Various phenomena like dynamic stalling, blade vortex interaction, blade-shaft wake interaction, and high noise generation account for the reduced efficiency of the turbine^8–14^. Hence, most research associated with VAWTs has been to increase the global efficiency of the turbine by suppressing or mitigating these losses. Dynamic stalling of VAWT blades is one of the most studied phenomena, with active research on controlling and mitigating the same using various techniques like flow control and augmentation devices.

Implementing VAWTs in a multi-rotor arrangement has also been beneficial, as the multi-rotor setup has been shown to provide higher power than a single rotor^15^. This can be attributed to the mutual synergetic interactions between the rotors. The higher efficiency of the multi-rotor system has been explicitly explored with a double-rotor arrangement, and results indicate that the performance of the pair of turbines is dependent on various geometrical parameters associated with the individual turbines, as well as the rotor spacing, angular positions, and direction of rotation of the double-rotor setup^15–22^. The co-rotating and counter-rotating rotor setup, where rotors are placed next to each other, has been explored more, compared to the contra-rotating setup, where the rotors are placed one above the other, along the same central shaft. The counter & co-rotating rotor setups of VAWTs have shown improved power performance by indicating a higher mean power coefficient and a smaller wake downstream^17,21,22^. The implementation of the aforementioned setup, if appropriately optimized in terms of rotor spacing, relative angular positions, and other properties, shows great potential for wind farm setups with higher power density than HAWT farms of similar scales^23^. While research on contra-rotating VAWTs is minimal, few studies indicate improved performance and reduced hysteresis on blades due to dynamic stalling^24–28^.

Flow control techniques, which include both active and passive flow control, focus on imparting momentum and energy to the boundary layer flow to suppress flow separation, reducing the impact dynamic stall has on the blades and reducing the hysteresis on the blades of the VAWTs. Flow control devices are also used to mitigate tip losses and reduce noise generation. Various flow control techniques like dimples, suction and blowing jets, leading and trailing edge serrations, gurney flaps, vortex generators and wingtip devices have been implemented on VAWTs and studied and have shown appreciable results in mitigating these losses and improving turbine efficiency^14,29–43^. External augmentation devices, which include guide vanes, diffusers, shrouds and wind-gathering devices^44–51^, are placed in the vicinity of the VAWT to augment the power generated by the turbine. All these external devices redirect the flow towards the rotor plane and augment the flow velocity, resulting in increased power production. In some cases, the velocity augmentation provided by these devices can also help suppress and mitigate dynamic stalling due to the blades experiencing a higher Reynolds number and improve the turbines’ starting torque^44,47,48,51^. Amongst the three mentioned external augmentation devices, the omnidirectional property of a VAWT is lost in the presence of a diffuser^49–51^, making it not sought after, especially for urban applications, where the wind direction varies with the infrastructure within the Urban Canopy Layer. Nevertheless, diffusers have proven to be an effective means to extract more power from VAWTs. Guide vanes, on the other hand, mainly focus on redirecting the incoming flow towards the rotor plane and only focus a little on the velocity augmentation. In addition, in the presence of wind-gathering devices, there exists a synergetic interaction between the blade tip vortices and the surface flow of the WGD, thus enabling the dissipation of the tip vortices into smaller vortices with lower energy and helping mitigate tip losses^45^. Between WGDs and guide vanes, WGDs are preferred in this study due to the simplicity of their design process, their ability to mitigate tip losses, and their ability to provide augmenting capabilities.

The Atmospheric Boundary Layer, which acts as a gradient, depends on the surface roughness^52–54^, and infrastructure in urban setups adds to the surface roughness and affects the ABL and the wind resources. With reduced surface roughness at higher altitudes^54,55^, the wind speeds and direction are less affected, providing a higher power generation potential. Airborne Wind Energy systems (AWEs) exploit the high wind speeds at high altitudes and harness power from the same^56^. This is generally done using tethered devices like kites, sails, gliders, aerostats and aircraft. AWEs forego the conventional usage of large towers and other support structures, thus also foregoing the usage of large plots of land which reduces manufacturing and maintenance costs. The various AWE systems can be classified into Ground-Gen and Fly-Gen AWEs. In the former, the power is generated by converting the mechanical energy produced due to the traction force transmitted from the airborne system to the ground-based generator. In the latter, the power is generated onboard the airborne system and fly-gen AWEs are known to carry the wind turbines with them for power production. Examples of ground-gen AWEs include the implementation of tethered inflatable kites, skysails, gliders and semi-rigid wings^57–63^. Examples of fly-gen AWEs include implementing wind turbines on platforms like aircraft, quadcopters and aerostats^64–70^.

The idea of designing an aerostat capable of lifting a wind turbine will be explored in this study. Aerostats have most commonly been used for surveillance and communications^71–76^, and implementing it as an AWE system has yet to be explored in depth. Aerostats are a cheaper alternative than heavier-than-air systems for such surveillance and last-mile communications applications^76^. Aerostats are filled with lighter-than-air (LTA) gases like hydrogen or helium, exploit aerostatic lift to carry the payload to the desired altitude, and are connected to the ground via tethers. Various aerostat profiles exist, with the most common being spherical, teardrop-shaped and spheroid-based aerostats. While the spherical-shaped aerostats are the simplest to design, the drag experienced by the spherical aerostat is very high, causing high amounts of lateral drift, which is called blow-by, which reduces the operational altitude of the system. While teardrop-based aerostats are highly efficient by providing aerodynamic lift in addition to the aerostatic lift, the requirement of rigid or inflatable fins for stabilization adds to the self-weight of the aerostat and reduces payload capacity^77^. On the other hand, spheroid-based aerostats like oblate spheroid based aerostats are far simpler in terms of design, produce lower drag than spherical aerostats, require extremely lightweight stabilization systems like kites and sails, and have been shown to outperform the other aerostats in the presence of heavy winds^76,77^.

It has been established that the implementation of aerostats for wind power generation needs to be studied more. In addition, no study to date exists to implement a VAWT, nonetheless that of a multi-rotor setup in an airborne platform. This study addresses these shortcomings and will provide a framework for the design and analysis of an airborne WGD-CR-VAWT system. The current study acts as a continuation of the previous study performed by the authors on the design of CR-VAWTs^28^. The novelty of this work would be providing a methodology for the design optimization of a WGD for a CR-VAWT, designed by the authors in the past study^28^. Additionally, the current study will also focus on the conceptual design of an aerostat, where the WGD-CR-VAWT designed would act as a payload, which would be elevated to an altitude via the designed aerostat. This study would provide a basis for the design of airborne VAWTs, something which has not been explored as much as airborne HAWTs and little to no literature on past studies associated with the former exists. The study as a whole can be divided into two parts, with the first detailing the conceptual design procedure for the airborne ducted CR-VAWT. The design optimization would be done using the Taguchi method and CFD analyses. This will be followed by the conceptual design of an oblate spheroid aerostat and tether profile estimation, which could lift the WGD-CR-VAWT system to a desired altitude. The study also includes a comparative analysis of the ducted and open airborne CR-VAWT performance and an analysis of both the airborne systems under skewed flow. The current study is purely a conceptual study showing the potential for an airborne WGD-CR-VAWT system that can be implemented in urban and rural setups as an airborne wind turbine, which can produce more power and more efficiently. Based on the results and limitations of the study, a framework for the design, analysis and implementation of such systems have been provided by the authors, which can act as a guide for studies associated with designing such systems.

The article is structured in the following format: “Methodology” explains the methodology implemented in this study for designing and assessing the airborne system. “Results and discussion” discusses the optimal design of the WGD and its properties, followed by the final sizing of the aerostat and the tether profile required to carry the WGD-CR-VAWT as a payload to the desired operational altitude. Additionally comparative analyses of the global performance of the airborne systems in both open and ducted configurations under ideal and skewed flow conditions are provided. “Limitations of current study and a proposed framework for future work on airborne WGD-CR-VAWT” discusses the limitations of the current study and provides a framework for future work on the proposed airborne system. “Conclusions and future work” summarizes the inferences made in this study and indicates the potential for future exploration of the proposed system.

## Methodology

### Geometric specifications of CR-VAWT

**Fig. 1:**
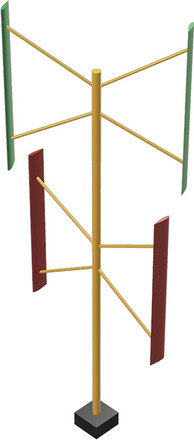
Contra-rotating VAWT.

The CR-VAWT used in this study was designed, analyzed and optimized by the authors as part of a previous study^28^. The study involved the design optimization of a CR-VAWT using the Taguchi method and CFD analyses. Results from the optimization study revealed a 14% increase in the power generated by the optimal CR-VAWT compared to the conventional VAWT at the optimal TSR. Additional details of the study and corresponding results can be found in Ref.^28^. The blades of the CR-VAWT comprise the NACA 0018 airfoil with a chord length of 0.12 m and a blade length of 2 m, respectively. Remainder details of the turbine are summarized in Table 1. A pictorial representation of the CR-VAWT is given in Fig. 1. The primary rationale behind the choice of the turbine being a CR-VAWT instead of a conventional VAWT was that the airborne system on which these would be deployed would not require additional anti-torque mechanisms, which would be required in the case of the latter.

**Table 1:** Geometric specifications of CR-VAWT.

Airfoil	NACA 0018
Number of blades (*N*_*b*_)	4
Turbine diameter (*D*_*t*_)	1 m
Blade height (*H*_*b*_)	2 m
Swept area (*A*_*s*_)	4.01 m^2^
Solidity (*σ* )	0.48
Chord length (c)	0.12 m
Axial gap (*A*_*g*_)	1 cm

### CFD modelling

Due to the current work being a continuation of the previous work by the authors, similar computational settings in terms of discretization methods, numerical schemes and turbulence modelling were chosen for this study^28^.

#### Computational domain

A transient analysis was implemented to study the system and accurately model the near field and farfield flow. For the CR-VAWT, the computational domain was split into a stationary domain and two rotating domains. Each rotating domain consisted of one turbine, and rotational velocity was imposed on them so that the direction of rotation of one turbine was opposite to the other, creating the contra-rotating effect. The stationary domain, which is cuboidal, measured 20D in the upstream & cross-stream directions and 40D in the downstream direction. The cylindrical rotating domain of each turbine measured 1.5D × 1.5H, respectively. These dimensions for the computational domain are required to properly model both the near and far wake flowfield. The following dimensions led to a blockage ratio, *B*_*R*_ = 0*.*5%, which is less than 3%; hence, no blockage corrections were included. The dimensions of the computational domain align with the guidelines published by Rezaeiha et al.^78,79^ on the simulation of VAWTs using CFD tools.

#### Mesh generation

VAWTs, being rotating bodies, require dynamic meshing strategies for their simulations. Both sliding mesh and overset mesh strategies have provided considerable success rates in terms of the overall accuracy of the results, with the former being the go-to approach for rotating bodies and the latter gaining importance in recent times. However, similar to the previous study, the overset meshing strategy is preferred over the sliding mesh due to the faster convergence, reduced computational time and increased solution accuracy offered by overset meshes^80^. In overset meshing, multiple near-body grids are overlapped on a singular background grid. The overlapping region is called the overset interface/boundary. The governing equations are then solved separately for both the background and the near body grids, and the solution is interpolated across the overset interface, respectively^81,82^.

**Fig. 2:**
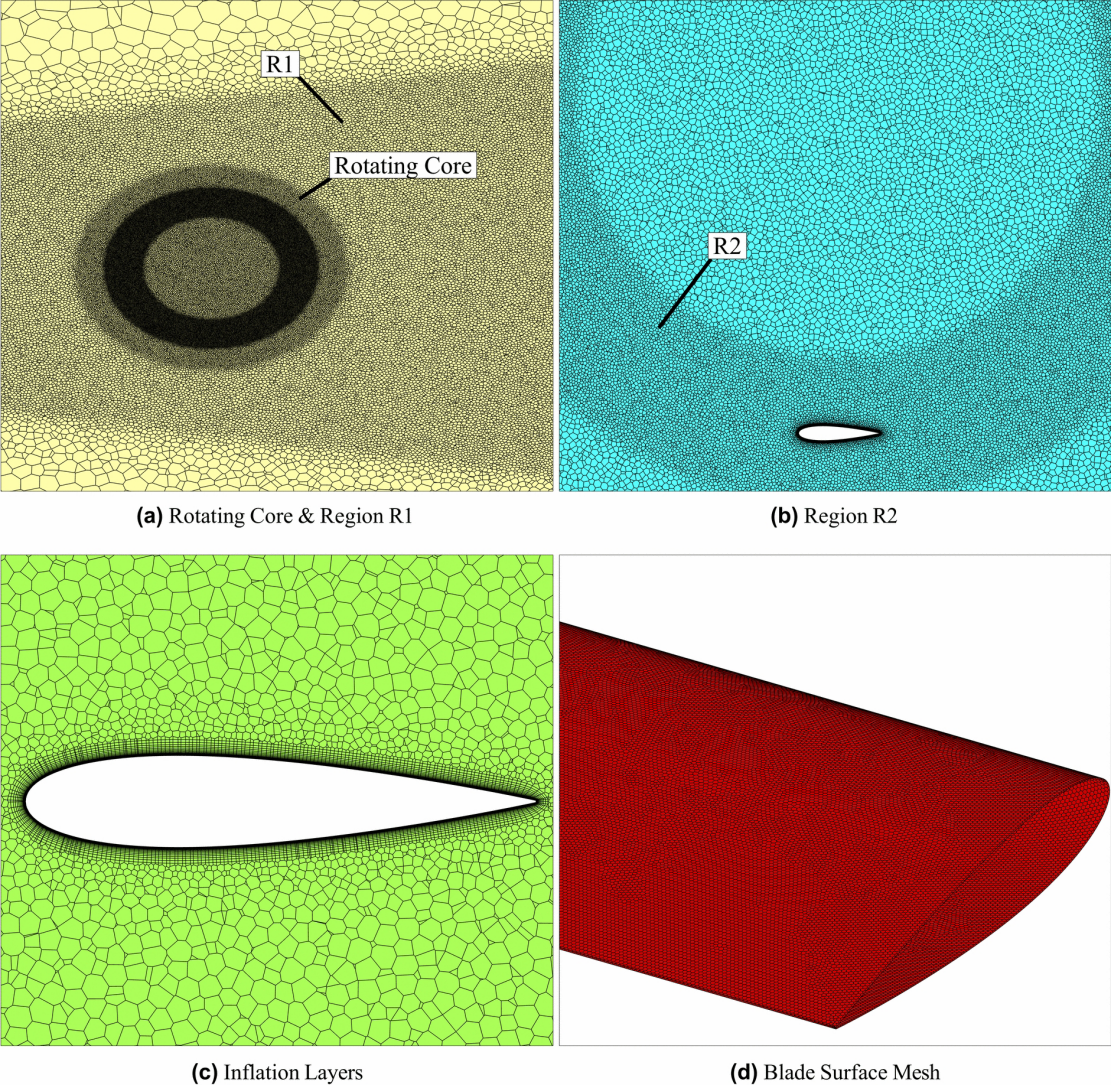
Schematic representation of the computational mesh.

As per the requirements of overset meshing, the stationary domain was chosen as the background mesh, and the two rotating domains of the CR-VAWT were chosen as the near-body mesh and made to overlap on the background mesh. The domains were discretized using polyhedral elements. Polyhedral elements generally coexist with multiple neighbouring cells and have six optimal flow directions, increasing the accuracy of the calculated gradients and producing good quality grids^83,84^. In addition, compared to a tetrahedral mesh, the cell count in a polyhedral mesh is lower, leading to faster computations. Two regions of local refinement, R1 and R2, were created in addition to the global mesh to model the near-field and farfield flow accurately. For the accurate capture of boundary layer flows, the *y*^+^ of the mesh was maintained at less than 1, which helps in the accurate modelling of the flow within the viscous sub-layer as well. Prism elements were added via the addition of 20 inflation layers to the walls of the bodies for the accurate modelling of the boundary layer flows. Schematics of the generated mesh at different locations are given in Fig. 2.

#### Turbulence modelling, numerical settings and boundary conditions

The complexities associated with the flow associated with VAWTs generally call for a high-order turbulence model for the resolution and modelling of the temporal and spatial scales of the said flow. LES-based simulations, which involve the spatial and temporal resolution of the large-scale eddies and modelling of the small-scale eddies via sub-grid scaling, while known to be very accurate, are computationally expensive due to the requirement of high-resolution grids. For this reason, Unsteady RANS (URANS) based models were chosen for the simulations. In RANS-based models, all eddies, irrespective of scale, are modelled via Reynolds Decomposition. While this leads to a drop in the level of accuracy as compared to LES simulations, high-resolution grids are not a pre-requisite for URANS-based simulations, hence saving computational time and costs, respectively. Due to the ability of URANS-based models to predict many primary flow features, they have been commonly used for VAWT simulations in the past.

The current study implements the Transition SST (TSST) model among the available URANS models. The TSST model is based on the k-*ω* SST model^85^, along with two additional equations which solve for the intermittency (*γ*) and the momentum thickness (*Re*_*θc*_), respectively. The TSST model is preferred over the two-equation k-*ω* SST model due to its ability to model and predict flow transition accurately. Additionally, one of the vital flow phenomena associated with VAWTs, dynamic stalling, is better predicted by the TSST model, as compared to the k-*ω* SST model, which indicates a merging of the Dynamic Stall Vortex (DSV) with the secondary vortex^86^. To accurately predict and model the transitional flow features and their properties, the equations governing intermittency and momentum thickness are tuned towards the incoming turbulence. Additionally, to avoid the over-prediction of the production term in the k-equation, turbulence limiters, as suggested by Kato and Launder^87^, have also been implemented. The TSST model has proven to be viable and useful in predicting transitional flows and flow separation-based phenomena not only in VAWTs in the past but also in other engineering applications, respectively^88–92^.

With regards to the numerical settings, for the pressure–velocity coupling, the coupled solver was implemented, which solves for the pressure and velocity together, simultaneously and achieving this implicit coupling via the implicit discretization of pressure gradient terms in the momentum equation, mass flux across cell faces and Rhie-Chow pressure dissipation terms, respectively. As for the spatial discretization, a least-cell-based model and second-order and third-order MUSCL schemes for solving the pressure and transport equations were chosen, respectively. The third-order MUSCL scheme combines the central differencing scheme and the second-order upwind scheme, which potentially increases spatial accuracy by reducing numerical diffusion. As for temporal discretization, a bounded second-order implicit scheme was implemented. The boundary conditions used to simulate the flow are summarized in Table 2. It is to be noted that the boundary conditions, except for the freestream density and dynamic viscosity, have been kept the same from the previous studies performed by the authors^28^.

**Table 2:** Boundary conditions.

Freestream velocity	9.3 m*/s*
TSR	0.5–5
Rotational velocity	9.3–93 rad*/s*
*Re*_*c*_	0*.*85 − 3*.*9 × 10^5^
Turbulence intensity	1%
*ρ* @ 100 m AGL (MSL = 920 m)	1.1025 kg/m^3^

### Convergence studies and model validation

Before proceeding with the simulations, a series of spatial and temporal convergence studies were performed to ensure proper spatial and temporal discretization methods and grids were implemented. Moreover, to check the accuracy of the CFD simulation, the numerical schemes, boundary conditions and turbulence models implemented, the results were validated against past studies. Simulations were run at *U*_∞_ = 9*.*3 m/s and *λ* = 2*.*5 for the convergence tests and the variation of the measured instantaneous moment coefficient acting on the blade (*C*_*m*_) with azimuth angle (*θ*) was reported. The variations in each convergence study between tests were reported in terms of the root mean square value (∆*C*_*m,rms*_), respectively. Any mention of the azimuth angle and the instantaneous *C*_*m*_ from here onwards will concern the blade of the upper turbine of the CR-VAWT, and the relative position of the lower turbine and its blades would be the conjugate of the azimuthal position of the upper turbine’s blades.

#### Spatial and temporal convergence studies

**Fig. 3:**
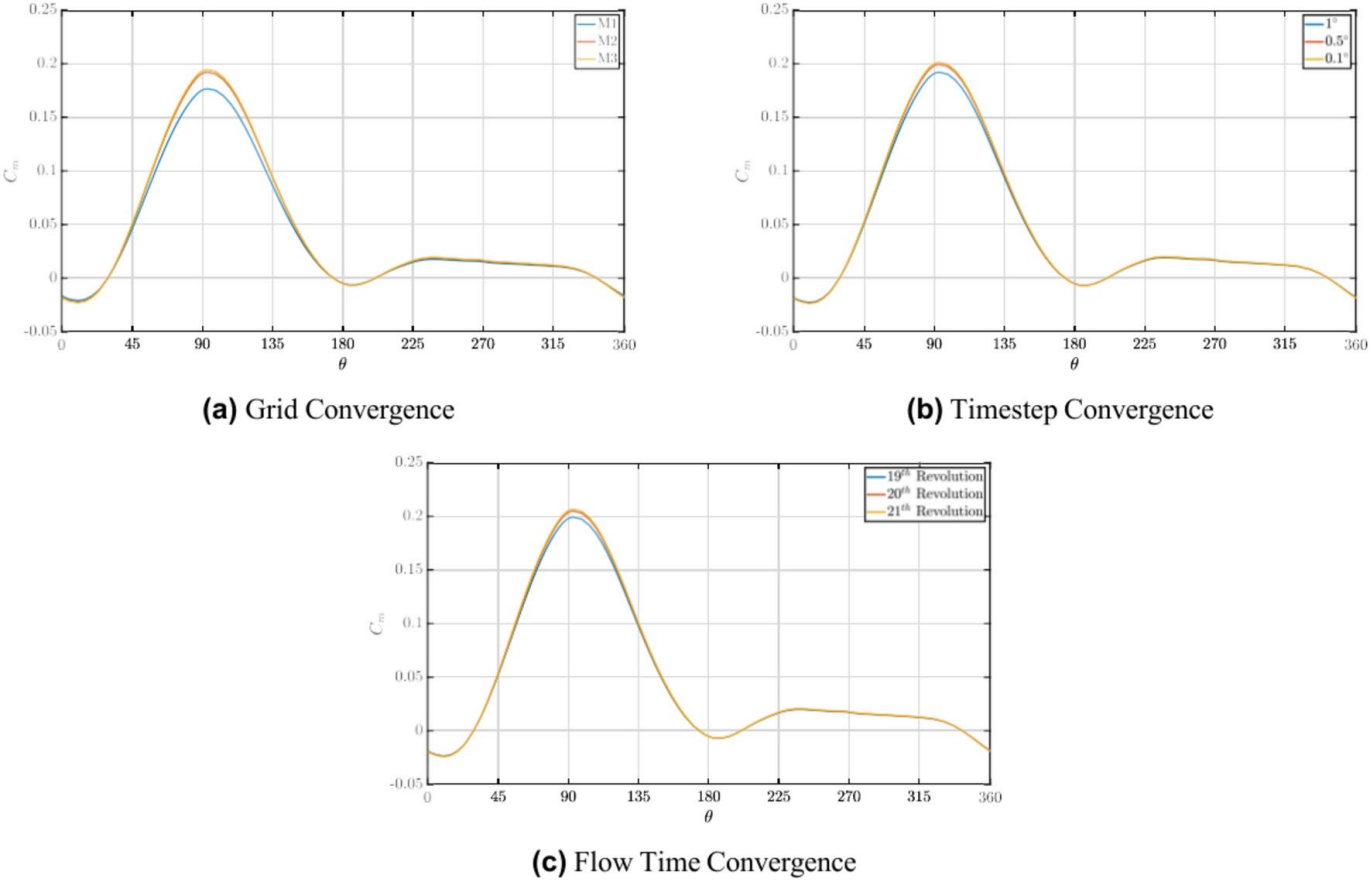
Sensitivity analysis.

For spatial convergence, a series of grid convergence tests were performed with three different grids with increasing elements. This was performed by decreasing the element size on the blade surface and the local refinement regions, R1 and R2, respectively. The *y*^+^ for the various grids were kept constant. Details of the different grids implemented in the study are reported in Table 3 and the *C*_*m*_* − θ* curves for the respective grids are depicted in Fig. 3a. Upon performing the analyses, a ∆*C*_*m,rms*_ of 8.71% and 1.3% was found between grids M1 & M2 and M2 & M3, respectively. The Grid Convergence Index (GCI), which verifies grid sensitivity, was found to be 1% for M2-M3, thus meeting the grid convergence requirements. Hence, grid M2 was implemented for further analyses.

**Table 3:** Details of grid convergence study.

Grid surface	No of elements	Element size on blade	Mesh skewness
M1	10*.*3 × 10^6^	1.5 mm	0.17
M2	27*.*6 × 10^6^	1 mm	0.13
M3	43*.*6 × 10^6^	0.5 mm	0.10

Two studies involving timestep size and flow time were performed for temporal convergence. For the convergence study associated with the timestep size, increments were given in terms of the azimuth angle per timestep (∆*θ*), which varied between 1°, 0.5°and 0.1°, and the corresponding *C*_*m*_* − θ* curves are represented in Fig. 3b. Upon studies, a ∆*C*_*m,rms*_ of 3.72% and 1.1% was found between ∆*θ* varying from 1° to 0.5°and 0.5°to 0.1°, respectively. Therefore, a timestep size corresponding to ∆*θ* = 0*.*5°was chosen for further analysis. Finally, concerning the flow time, variations regarding convergence were studied between complete revolutions of the turbine. *C*_*m*_* − θ* curves for the 19th, 20th and 21st revolution of the turbine is presented in Fig. 3c. Upon comparison, between the 19th and 20th revolution, a ∆*C*_*m,rms*_ of 2.75% and a ∆*C*_*m,rms*_ of 1.01% between the 20th and 21st revolution was observed, respectively. This indicated that the results showed little variation beyond 20 revolutions,

indicating a convergence condition with respect to the flow time of the simulation.

#### Model validation

**Fig. 4:**
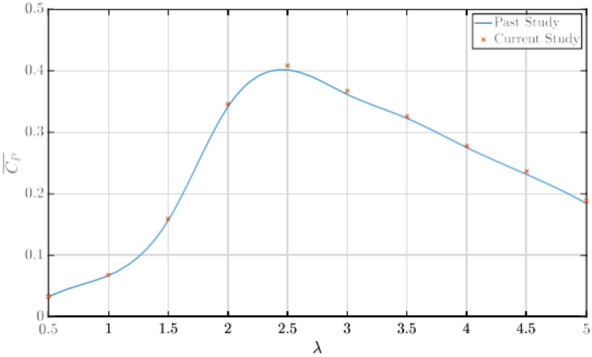
Comparison of *C*_*P*_* − λ* values between current study and past study^28^.

To validate the current numerical settings, schemes and turbulence models, the turbine was simulated for a range of TSRs between *λ* = 0 − 5, and results were compared with the past study performed by the authors^28^. Comparison of the *C*_*P*_* − λ* curves for the specified range of TSRs was used as the basis for the model validation, which is given in Fig. 4. A comparison of results showed that the *C*_*P*_ values matched closely with the previous study, and the *C*_*P*_* − λ* polar followed a similar trend. Minor deviations ranging between 1.1 and 3.15% were found between the results compared, with an average deviation of 1.8% between all the TSRs tested. This deviation in the results can be attributed to the difference in the number of elements in the grid, and numerical settings, which implements a higher-order model for the spatial discretization of the transport equations, compared to the second-order upwind model implemented in the previous study and the simulations being run for 20 turbine revolutions, compared to the 15 turbine revolutions run in the previous work. Nonetheless, these variations are within acceptable range and hence validate the methodologies implemented in the CFD simulations.

Moving forward, all simulations were performed using the grid M2, with a timestep size corresponding to ∆*θ* = 0*.*5°and run for 20 turbine revolutions, respectively. All calculations were parallelized over two separate workstations. The first workstation included an Intel i9-13950HX processor containing 24 cores, with a clock speed of 2.2 GHz, boosted up to 5.5 GHz, a 2 × 64 GB SODIMM DDR5 RAM and 12 GB NVIDIA A4000 graphics card. The second included an Intel Xeon w3-2423 processor with 6 cores and a clock speed of 2.1 GHz, boosted up to 4.2 GHz, a 256 GB SODIMM DDR5 RAM and 8 GB NVIDIA T1000 graphics card, respectively. All simulations were run in ANSYS 2023R2.

### Design optimization of WGD: Taguchi method and CFD

As discussed in “Introduction”, to augment the incoming flow velocity towards the rotor, a WGD is preferred over other flow augmentation mechanisms, mainly because the omnidirectionality of the VAWT is not lost in the presence of WGD. The main aim of the WGD is to augment the flow velocity at the rotor plane so that the blade experiences higher incoming velocity, which helps in increasing the overall power output and has a local effect on the blade loading, for example, delay or reducing dynamic stall, since the blades would be experiencing a higher Re, compared to the blades on a bare turbine. A baseline ellipsoid shape was chosen to proceed with the design optimization of the WGD. An ellipsoid-shaped WGD is preferred since the study focuses on implementing it as an airborne system. The ellipsoid contributes to less drag than other spheroid-based volumetric shapes. Additionally, while other shapes can be used to provide lesser drag, the ellipsoids implemented as the WGD, in addition to augmenting the flow velocity, can also be designed and optimized in a way to contain LTA gas and provide some percentage of aerostatic lift, which in turn can reduce the size of the main aerostat needed to carry the whole system. The latter idea is just a proposal, must be investigated further, and will not be considered in this study. As a part of this study, the sole purpose of the WGD would be to provide flow augmentation to the CR-VAWT, respectively. The aerostat will provide the aerostatic lift to keep the system airborne, whose sizing and design will be discussed in the forthcoming sections.

Proceeding with the ellipsoid-shaped WGD, the Taguchi method, along with CFD, was implemented to find the optimal design of the WGD. The Taguchi method is a widely used optimization method which focuses on minimizing the quality loss in an experiment. The Taguchi method was implemented in the previous study by the authors to find the optimal design of the CR-VAWT. Details of the Taguchi method and its implementation can be found in Ref.^28^. With the aim of the WGD being power augmentation, the augmentation ratio, *ε*, is chosen as the target parameter, and the aim is to find the optimal WGD design, which provides the maximum power augmentation. The S/N ratios are expressed in terms of larger the better, which yields the combination of design parameters with the highest value of *ε*. The augmentation ratio is the ratio between the average power coefficients of the ducted and open CR-VAWT configurations and is given by

1 $$\varepsilon = \frac{{{\text{CP}},{\text{ducted}}}}{CP,open}$$

#### Design factors and orthogonal array

For the Taguchi analysis, three different design parameters were chosen: ellipsoid semi-major axis, semi-minor axis and tip gap between the blade and the WGD. These three parameters were selected since the dimensions of the semi-major and semi-minor axis would govern the curvature of the WGD, which in turn governs the augmentation property of the WGD by redirecting a larger mass flow of air through the rotor plane. In addition, the tip gap between the blades and the WGD is essential since the interaction of the tip vortices with the boundary layer of the WGD can show promising results in tip loss reduction, thereby increasing the efficiency of the turbine. Based on this, a three-factor design table was generated, and the various design levels of the different parameters tested are mentioned in Table 4. Based on the design factors provided, an L9 (3^3^) orthogonal array was created, in which each test case consisted of the best representation of the combination of parameters from the universal set of combinations. Nine test cases (henceforth denoted as experiments E#) were selected and are presented as part of the OA in Table 5. CFD analyses were run for each of these experiments, and the value of *ε* was calculated for the *S/N* analysis, which resulted in the optimal design of the WGD. In addition to the *S/N* analysis, the weightage of each design factor on the value of *ε* is also calculated to find which design parameter tends to affect the augmenting capability of the WGD respectively.

**Table 4:** Design factors and levels for WGD optimization.

Factors	Parameters	Notations and units	Levels		
			1	2	3
A	Semi-major axis	a (*m*)	0.75	1	1.25
B	Semi-minor axis	b (*m*)	0.25	0.5	0.75
C	Tip gap	t (*m*)	0.075	0.05	0.025

**Table 5:** L9 (3^3^) orthogonal array.

Experiment	Factors (coded)			Factors (uncoded)		
	A	B	C	a (*m*)	b (*m*)	t (*m*)
1	1	1	1	0.75	0.25	0.075
2	1	2	2	0.75	0.5	0.05
3	1	3	3	0.75	0.75	0.025
4	2	1	2	1	0.25	0.05
5	2	2	3	1	0.5	0.025
6	2	3	1	1	0.75	0.075
7	3	1	3	1.25	0.25	0.025
8	3	2	1	1.25	0.5	0.075
9	3	3	2	1.25	0.75	0.05

### Aerostat sizing and tether profile estimation

As discussed in “Introduction”, aerostats are platforms which provide aerostatic lift via the use of Lighter-Than-Air (LTA) gases like hydrogen or helium and are used for various applications. The conceptual design and sizing of the aerostat start with the desired weight of the payload to be carried and the shape of the aerostat envelope. Based on this, assumptions are made accordingly to arrive at the size of the aerostat required to carry the specified payload, the individual mass-breakdown of each component of the aerostat, and finally, obtaining the overall static lift produced by the aerostat, which is a function of the buoyant force and the self-weight of the aerostat, respectively. The design process is highly conservative, and the final design is obtained via an iterative approach. This acts as a starting point for the design of any aerostat to obtain the its initial sizing parameters, after which the design process like with any other product follows an iterative procedure, with changes and refinement towards the final design.

Amongst the various aerostat profiles discussed in “Introduction”, the oblate spheroid-based profile was chosen for this study. The sizing of an oblate spheroid aerostat begins with taking inputs of the payload mass, Mean Sea Level (MSL), operational altitude above MSL, atmospheric conditions at operational altitude, choice of LTA gas and its properties, choice of material for aerostat envelope and tether, and its properties, inflation ratio and many more. While not all material properties are required in the initial design stage, the specific mass of the tether and the aerostat envelope are critical factors in determining the individual weights of the aerostat envelope and the tether, respectively. Once these inputs are available, the final size of the aerostat required to carry the specified payload is obtained via an iterative approach.

**Fig. 5:**
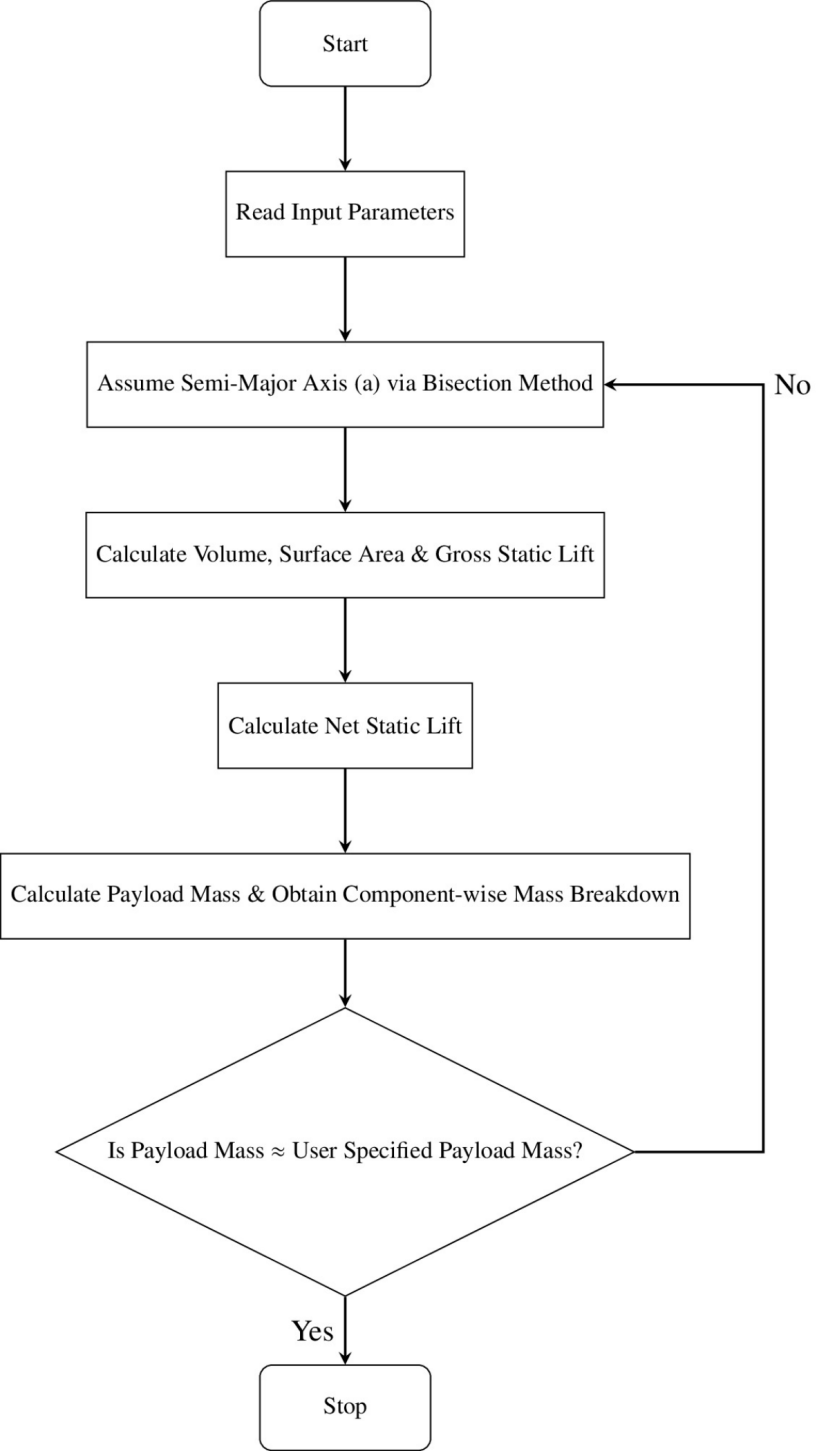
Flow chart of the iterative code.

This iterative approach begins by assuming the value of the semi-major axis (*a*) of the oblate spheroid, for which the volume and surface area are obtained. Once these values are obtained, using the Force-Weight approach^93^, the total buoyant force and the net static lift are obtained. The weight of the LTA gas to be carried to provide said static lift is also obtained in this step. With these values, the mass breakdown of individual components of the aerostat is obtained, and the final payload mass, which can be carried for the assumed value of *a*, is obtained. This is compared with the payload mass specified as input by the user, which must be equal. Until this criterion is met, this iterative loop keeps running, and upon meeting the required convergence criterion, the output provided includes the final dimensions of the aerostat, the individual mass breakdown of each component, and the net static lift as a function of the total buoyant force, respectively. Mathematical calculations associated with the Force-Weight method and mass calculations of individual components are summarized in A. The bisection method was implemented to reduce computation time. The initial value of *a* was assumed from a large interval of values between [0,1000 m], and the convergence criterion for the code was kept as 1e-6. A flowchart of the same is provided in Fig. 5

To remain conservative towards the design process, a few choices and assumptions were made, which were necessary to be included and accounted for, and are listed below:

- *Material properties* The material properties, mainly the specific mass of the materials used for aerostat envelopes and tethers, were taken directly from readily available data regarding the same^94^.
- *Choice of lifting gas and purity* Helium, although an expensive choice, was chosen as the lifting gas of choice due to its inert nature and easier handling compared to hydrogen, which is flammable. In a realistic scenario, 100% pure helium cannot be manufactured, and the gas is prone to contain some percentage of impurities; hence, the gas was considered to be 96% pure for this study. The purity of the lifting gas is known to have a direct effect on the net static lift provided by the aerostat.
- *Free lift* A 15% free lift is added to the total buoyant force generated by the aerostat to withstand turbulence, downdrafts, weather changes, maintenance of operational altitude and proper tether tension to avoid slacking of tether on the ground due to low tension^95^.
- *Superpressure and superheat* Superpressure and superheat are the differential pressure and temperature between the external atmosphere and the interior of the aerostat. Superpressure accounts for the rigidity of the aerostat envelope under static and dynamic conditions, and studies show a 1–3 inches water gauge must be maintained for the hull rigidity^95^. Superheat, on the other hand, if positive, enables the expansion of the gases within the aerostat and enables the production of higher static lift, and negative superheat, also called supercool, produces the opposite effect. For this study, a value of 5 K was used for superheat.
- *Additional weight considerations* While the design process gives the output of the mass of the key individual components, data regarding various other components are not readily available at this stage of the design. To account for these, some assumptions are made and incorporated into the calculations of the current design process. Key assumptions made are:
- An increment of 15% to the envelope weight to account for seams and overlaps.
- A 30% increment in tether length and its corresponding weight to account for the drift and blow-by of the aerostat due to ambient winds and maintain its position at operational altitude.
- The combined mass of various miscellaneous components like payload recovery devices^96^, rapid deflation systems^97^, hooks, stabilization mechanisms (active/passive) and many more were considered to be 20% of the overall deployable payload mass.

As for the stabilization of the aerostat, oblate spheroid aerostats are most commonly equipped with sails or kites^77^, which was not part of the design process at this stage since various values associated with the dynamics of the system are not present at this stage. Based on all the inputs, choices and assumptions made, the final size of the oblate spheroid aerostat is obtained from the code, which can lift the WGD-CR-VAWT system above ground levels for higher power outputs.

Once the aerostat sizing is completed and the net static and gross lift are obtained from the above iterative code, the tether profile can be estimated. Two forces act on the tether of the aerostat: the tether tension and the drag force. The estimation of the tether profile and the overall blow-by due to ambient winds are done by discretizing the tether into small elements. Each of these elements is under the influence of drag force, tensile force and the individual weight of the element. Assuming the tether to be a rigid body and ignoring viscoelastic effects, the tension and the angle at the confluence point are used to calculate the tension and angle on the subsequent tether elements. This method of estimation of tether profile has been implemented in past studies, and the mathematical formulation of this method is provided in Ref.^72,98,99^.

## Results and discussion

### Optimal design of WGD

**Fig. 6:**
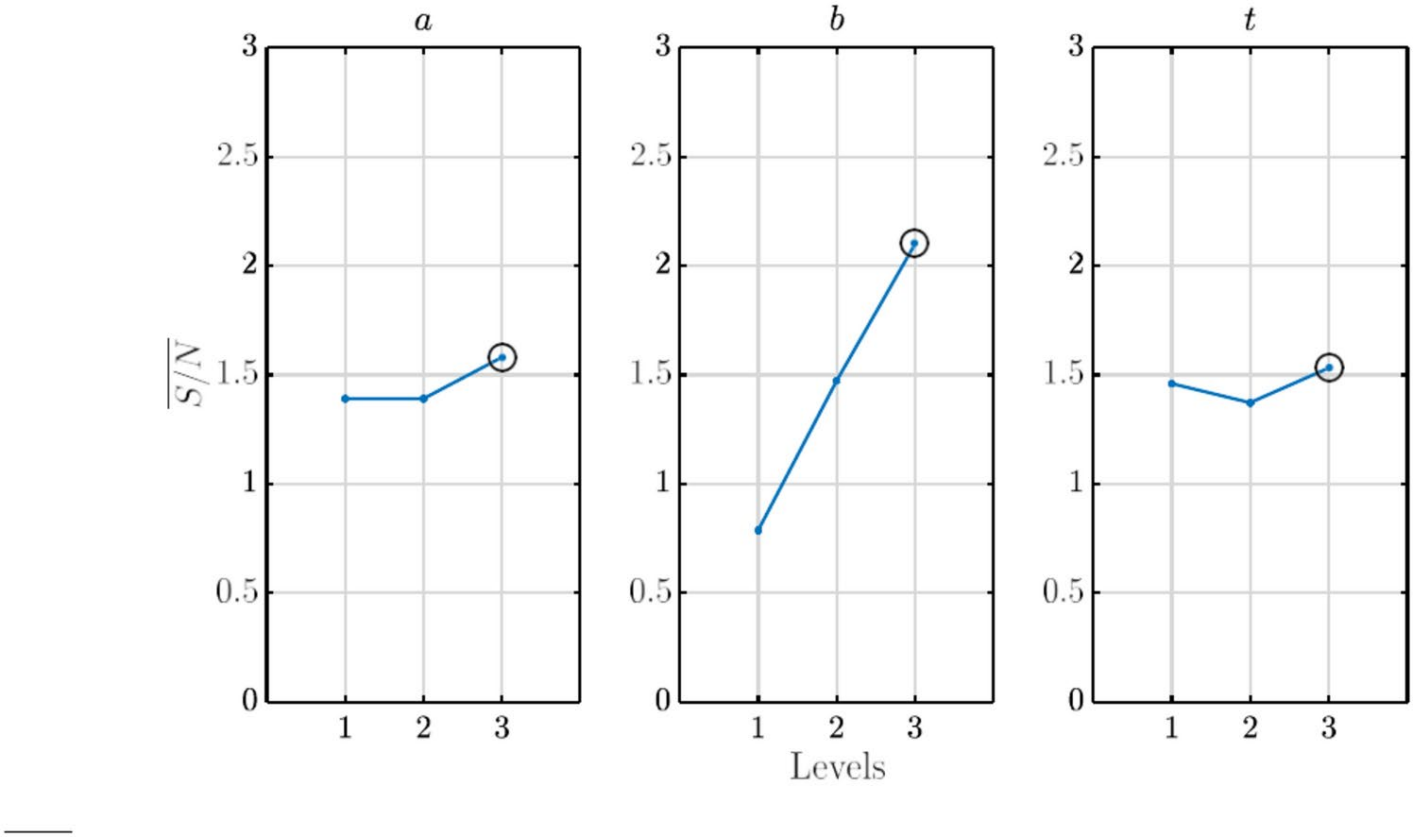
*S/N* values of design factors at different levels (black circles indicate the optimal value of each design factor).

**Fig. 7:**
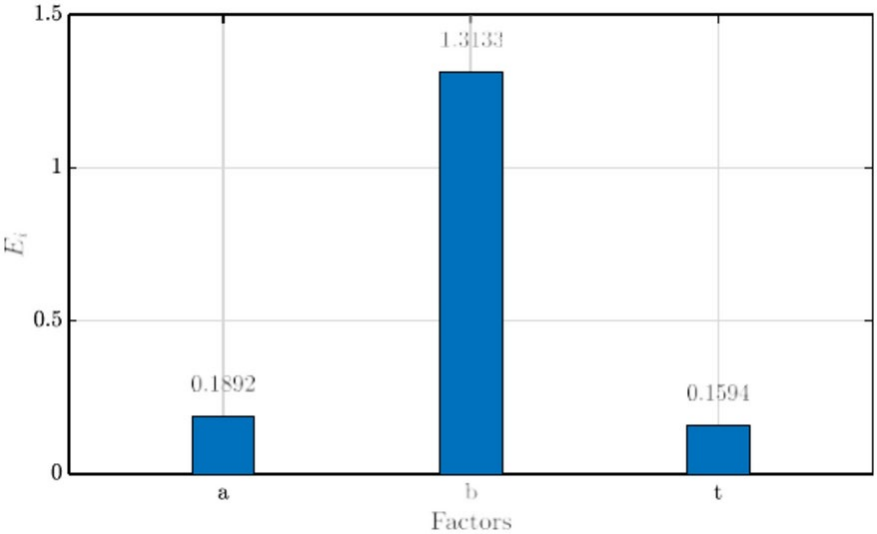
Extent of impact/weightage of design factors on *ε.*

CFD analyses were performed for all the nine experiments presented in the L9 orthogonal array in Table 5. In each case, the simulations were performed for *λ* = 2 − 4. The required mathematical calculations and formulae required for the *S/N* range analysis, which provides the mean value of the *S/N* ratio for each design factor at a given design level and the extent of impact analysis, which provides the weightage of each design factor towards the optimal design/performance variable has been provided in Ref.^28^. The values of the max attained *ε*, and the *S/N* ratios for each experiment are presented in Table 6. With these obtained values, performing the range analysis leads to the mean values of the signal-to-noise ratios, *S/N*, which are presented in Fig. 6. Additionally, the impact/weightage of each design factor on the optimal design of the WGD, as obtained from the extent of impact analysis, is also produced in Fig. 7.

**Table 6:** Summary of max attained *ε* and *S/N* from Taguchi analysis.

Experiment	*ε*	S/N
1	1.091	0.7565
2	1.1666	1.3384
3	1.2701	2.0768
4	1.072	0.6039
5	1.1905	1.5146
6	1.2666	2.0528
7	1.1221	1.0006
8	1.1977	1.5670
9	1.284	2.1713

From Fig. 6, the range analysis provides the optimal combination of design factors and their respective levels for the optimal design configuration of the WGD, which provides maximum power augmentation. Given that the goal of the optimization is larger the better, the combination of the levels of each design factor, which produces the highest value of *ε*, is circled in Fig. 6, respectively. In addition, the combination of levels of the design factors, which provide the best and worst case scenarios and their respective augmentation ratios, are summarized in Table 7. It can be observed that even the worst-case design of the WGD obtained from the Taguchi optimization provides up to a 5.1% augmentation in power, and the optimal design configuration of the WGD leads to a 34.7% increase in the power output, respectively.

**Table 7:** Best and worst configurations of WGD.

Case	a (*m*)	b (*m*)	t (*m*)	*ε*
Best case	1.25	0.75	0.025	1.347
Worst case	0.75	0.25	0.05	1.051

In addition to the range analysis, the extent of impact analysis (Fig. 7) revealed that the semi-minor axis (*b*) had the most effect/impact towards the optimization, followed by semi-major axis (*a*) and the tip gap (*t*). Quantitatively, each of the above-mentioned parameters exercised values of 1.3133, 0.1892, and 0.1594, respectively. The value of *b* takes more importance towards the final optimal design since, for a given value of *a*, it governs the curvature of the WGD, which provides a Venturi effect, thus increasing the mass flow of air through the rotor plane and increasing the augmentation ratio. Given that the target of the optimization process was the global augmentation of the power produced by the CR-VAWT, the impact of *t* on the optimization is as expected since the primary focus lies on global power augmentation and is not specific to tip loss reduction.

### Final aerostat sizing and tether profile

#### Final aerostat sizing

With the WGD optimized, the whole WGD-CR-VAWT system would act as the payload, which can then be carried by an oblate spheroid aerostat to higher altitudes above ground levels for increased power output. As discussed in “Design optimization of WGD: Taguchi method and CFD”, the ellipsoid-shaped WGD will not be used to provide aerostatic lift but could be considered in the future. The estimation of the payload mass, which would be the overall mass of the WGD-CR-VAWT system, was obtained from CAD software by including material properties in different components of the system. The blades and the WGD were considered hollow and made out of Carbon Fibre-Reinforced Polymers (CFRP), and the connecting elements like shafts and struts were composed of aluminium. Based on this, the CAD software provided a mass of 40 kg for the whole system. To remain conservative, an additional 10 kg is added to the overall payload mass to account for other mechanical structures, gearboxes and many more whose data are not present at this stage of the study.

Composites are used for wind turbine blade manufacturing due to their high strength-to-weight ratio and the requirement for structural rigidity during operation. The use of metal-based materials for connecting elements is due to the load-bearing capacity of metals and resistance to wear & tear during turbine operations. This choice of materials is purely for the initial estimation of the payload mass. It must be revised based on the structural analysis of the payload components, which would provide more accurate data on the material properties of the payload. With the payload mass obtained, the various inputs to be provided in the iterative code are summarized in Table 8. Additionally, in this case, only 95% of the aerostat is assumed to be filled with helium to account for the expansion of the LTA gases within the envelope due to diurnal changes in temperature and pressure. The specific weights of the tether material and the aerostat envelope material are directly taken from past studies.

**Table 8:** Inputs for aerostat sizing code.

Operational altitude AGL (m)	100
Mean sea level (m)	920
Payload mass (m)	50
Specific weight of tether (kg/m)	0.05
Specific weight of envelope (kg/m^2^)	0.2
Fineness ratio of oblate spheroid (−)	2.5
Inflation ratio (−)	0.95
Purity of LTA gas (−)	0.96
Superpressure (mmH_2_O)	50
Superheat (K)	5
Relative humidity (−)	50%
Relative density of water vapour (−)	0.622
Relative density of LTA gas (−)	0.1382

Upon running the iterative code with the given inputs, the outputs obtained are the individual mass of each component of the aerostat, along with the net static lift and the sizing of the aerostat required to lift the said amount of payload. Outputs obtained from the iterative code are summarized in Table 9. The outputs presented in Table account for the increments assumed in “Aerostat sizing and tether profile estimation”. This code can also be used to study and analyze the effect of various input parameters on the overall sizing and the net static lift generated by the aerostat by changing the input values accordingly to observe the variations.

**Table 9:** Outputs of aerostat sizing code.

Final semi-major axis (m)	3.802
Final semi-minor axis (m)	1.5208
Volume of oblate spheroid (m^3^)	92.082
Surface area of oblate spheroid (m^2^)	115.665
Mass of envelope (kg)	26.6
Mass of tether (kg)	6.5
Mass of LTA gas (kg)	16.55
Mass of Balloonet (kg)	5.033
Mass of miscellaneous items (kg)	12.5
Gross static lift (N)	1149.25
Net static lift (N)	937.55

**Fig. 8:**
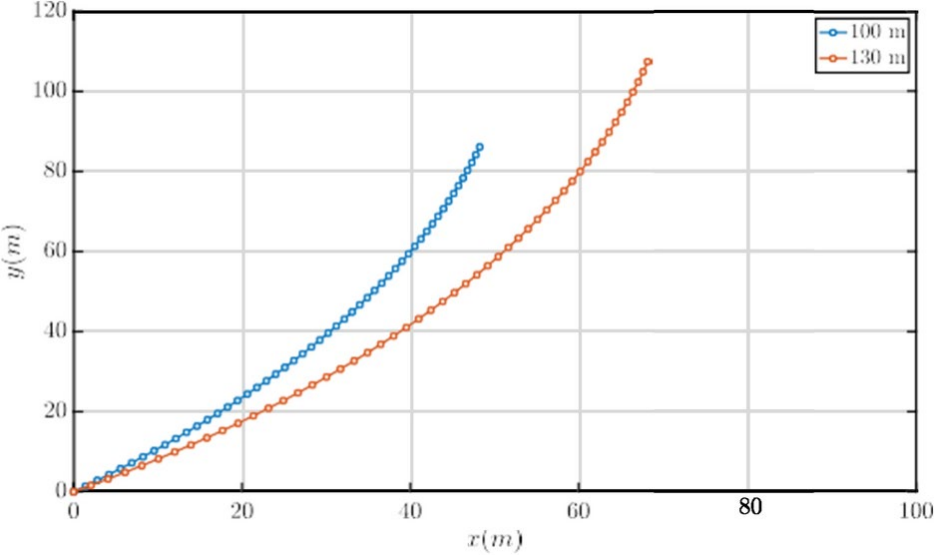
Tether profile @ *U*_∞_ = 9*.*3 m/s.

The net static lift obtained from the iterative code for the conceptual design of the aerostat can be used to obtain the tether profile in the presence of ambient wind. Figure 8 represents the tether profile for two different tether lengths. The curvature in the tether profile is primarily due to the drag force acting on each of the tether elements, and the drag coefficient of the tether element is assumed to be 1. Two cases are presented in Fig. 8 for a freestream velocity of 9.3 m/s. In the first case, the tether length is taken the same as the operational altitude AGL, which is 100 m, and in the second, a 30% increment as discussed in “Aerostat sizing and tether profile estimation” is provided. It can be seen that in the presence of ambient winds, due to blow-by, in the first case, the operational altitude of the aerostat is reduced almost by 30 m, which can reduce the effectiveness and the efficiency of the whole system, which would combine both the payload and the aerostat in this case. Accounting for the increment in the tether length, it can be seen that the system is maintained close to the operational altitude, and the excess tether helps account for the blow-by effects. Hence, excess tether is required to account for the blow-by effects.

### Performance comparison: open and ducted CR-VAWT

**Fig. 9:**
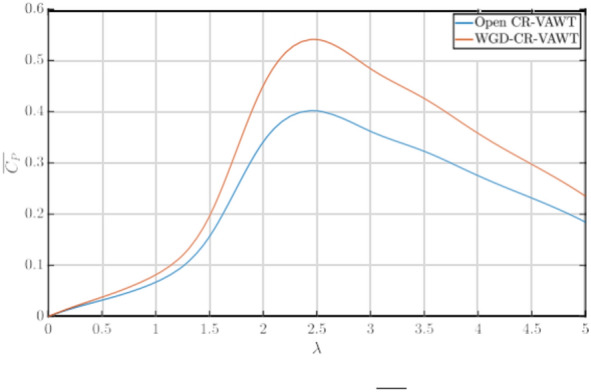
Comparison of *C*_*P*_* − λ* Polars.

As established earlier, the presence of a WGD helps augment the power generated by the CR-VAWT. Simulations were run for the CR-VAWT equipped with the optimal WGD, and the global performance of the turbine was compared with the open turbine and is presented in Fig. 9 in terms of the *C*_*P*_* − λ* polar. It is to be noted that all the simulations reported are run in the airborne configuration, i.e. in the case of both the open and WGD-CR-VAWTs, the turbine is elevated using an aerostat to a specified altitude as reported earlier. From the Fig. 9, it can be observed that the WGD provides an augmentation in the *C*_*P*_ at all tested TSRs. The highest augmentation is seen between *λ* = 2 − 4, with an average augmentation of 32% in the *C*_*P*_ values. The highest augmentation in *C*_*P*_ is obtained at *λ* = 2*.*5, with an augmentation of 34.5%. While augmentation in *C*_*P*_ is observed at all TSRs, they are not uniform. An average of 17% augmentation is observed at low TSRs. This can be attributed to the heavy losses experienced due to dynamic stalling at low TSRs. Similarly, as the TSR increases, while dynamic stall does not exist, losses due to the high rotational speeds of the turbine reduce the augmenting capability of the WGD, producing only a 27.7% augmentation in the *C*_*P*_. Hence, the optimal performance range of the WGD-CR-VAWT system is between *λ* = 2 − 4, with max *C*_*P*_ observed at *λ* = 2*.*5.

**Fig. 10:**
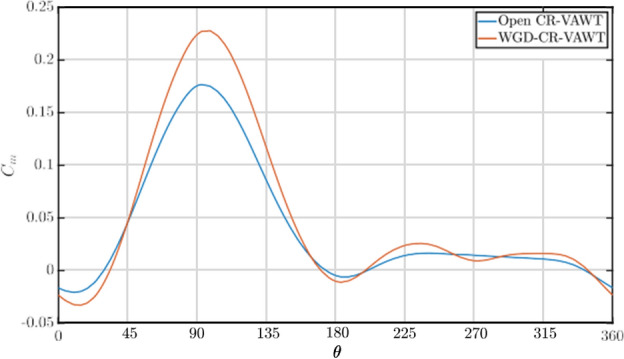
Comparison of Instantaneous *C*_*m*_* − θ* Polars @ *λ* = 2*.*5

To support the global performance of the turbine, it is also necessary to understand the local variations of different parameters. The instantaneous moment acting on the turbine blade is a useful local parameter that can help analyze the cyclic loading on the blades. Figure 10 represents the variation of instantaneous *C*_*m*_ with azimuth angle, *θ* at *λ* = 2*.*5. The WGD-CR-VAWT experiences a higher torque in the upwind direction (0° ≤ *θ* ≤ 180°) due to the blades experiencing higher velocities at the rotor plane due to the augmentation provided by the WGD, leading to an average of 41% augmentation in the torque experienced by the blades. While not much difference is observed in the values of torque in the downwind section between the blades of an open and WGD-CR-VAWT, an average augmentation of 20% in the torque values were observed.

**Fig. 11:**
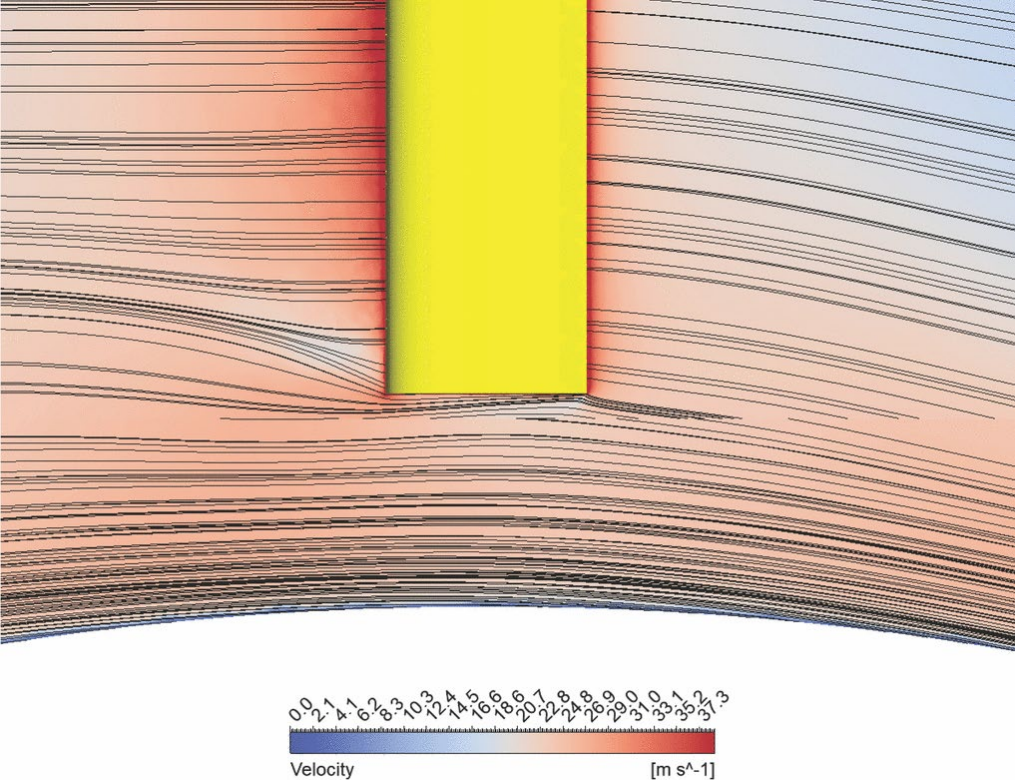
Blade tip-WGD interaction @ *λ* = 2*.*5

Finally, concerning dynamic stalling, while the blade still experiences the phenomenon, the blades experience a higher torque, thus generating higher power. While the WGD is not beneficial in suppressing or delaying dynamic stall, its augmenting capability helps produce higher torque, even in the presence of dynamic stall, thus increasing the turbine efficiency, even under the influence of dynamic stall. The WGD, however, is beneficial in reducing the tip losses experienced by the turbine. The low tip gap between the blade and the WGD surface helps in the local augmentation of the velocity near the tips, which reduces the tip losses. There exists a synergetic interaction between the tip vortices and the surface flow of the WGD, as indicated in Fig. 11, which shows the absence of circulation regions between the blade tips and the WGD, mitigating tip vortices, and increasing the overall efficiency of the system. The suppression of these coherent vortices is a possible indicator towards tonal noise reduction. However, proper aeroacoustic analysis and sound pressure level comparison is required to confirm the same and is not of concern with the current study. This is important as noise regulation would be a primary concern while deploying this system in urban environments.

### Airborne WGD-CR-VAWT under skewed flow: a preliminary study

**Fig. 12:**
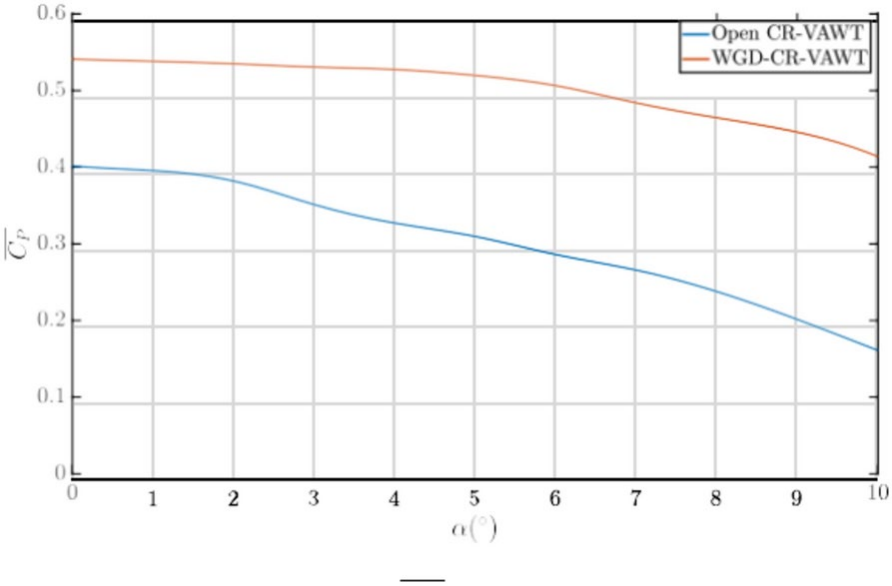
*C*_*P*_ vs *α* @ *λ* = 2*.*5

In the absence of data associated with the aerostat’s static and dynamic stability parameters, it is impossible to obtain the turbine’s performance data and its dependence on the dynamics of the aerostat. Additionally, the rotation of the turbine could affect the dynamics of the aerostat and, in turn, the whole airborne system. Hence, as a preliminary study, one of the parameters that can be studied is the aerodynamic angle of attack of the airborne system. It is known that aerostats operate under a small aerodynamic angle of attack, which provides a degree of aerodynamic lift. Assuming that the WGD-CR-VAWT is rigidly connected to the aerostat and does not move independently of the aerostat, we can assume that the rotor plane also experiences the same inclination. Hence, a simple study for *α* = 0 − 10°at *λ* = 2*.*5 was performed for the complete airborne system to understand the performance of the turbine in the presence of a skewed flow. The open CR-VAWT was also exposed to the same treatment to compare the performance of both turbines. Results of the obtained *C*_*P*_ are presented in Fig. 12.

From Fig. 12, it can be seen that the presence of WGD as a part of the airborne system is greatly beneficial when experiencing skewed flow. The presence of a WGD helps provide a sufficient degree of augmentation capability to the turbine, even under skewed flows. As the value of AoA increases, the augmenting capability of the WGD is also shown to reduce by providing an average augmentation of 17% beyond *α* = 5°. Additionally, even at a highly skewed state of 10°, the WGD-CR-VAWT produces 58% more power than the open CR-VAWT under the same conditions. This is still an appreciable percentage of augmentation provided by the WGD, compared to the open turbine, which shows a significantly decreasing trend in the value of *C*_*P*_ beyond *α* = 2°. In contrast, such a significant decrease for the WGD-CR-VAWT is observed only after *α* = 5°. This indicates that the airborne system, when equipped with a WGD, can still produce power more efficiently, and this can act as a basis to understand to what extent the system can experience a variation in the AoA to produce power efficiently. While this does not provide a basis for the complete dynamics of the system, it can act as a limiter to future studies which would account for the dynamics of the system.

## Limitations of current study and a proposed framework for future work on airborne WGD-CR-VAWT

**Fig. 13:**
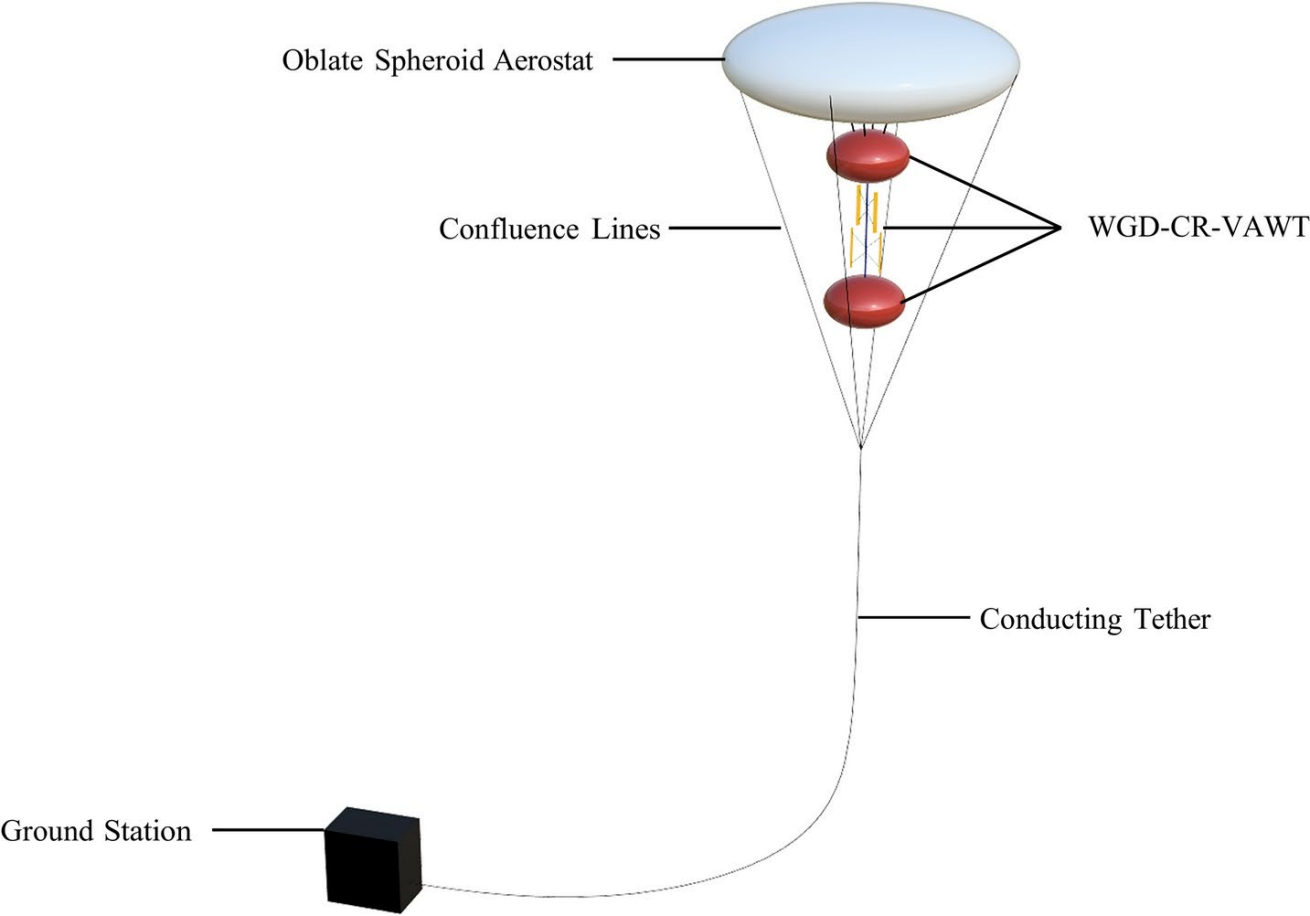
Artistic Render of the Airborne WGD-CR-VAWT^100^.

An artistic render of the Airborne WGD-CR-VAWT system is provided in Fig. 13. It is to be noted that various elements like the confluence lines, tether profiles, and ground station are purely for representation purposes only and are not to scale.

### Limitations

The current study focuses on the implementation of such a system and indicates its efficiency and effectiveness towards being developed into a fully functioning system. While the current study provides a basis for studying the concept of an aerostat-based airborne wind energy system using augmented CR-VAWTs, there are some limitations that must be addressed in the future. The following are some of the key limitations in this study:

- The optimization of the WGDs uses the Taguchi method, which is a singular target function-based optimization method. Multi-objective design optimization algorithms must be used in further studies.
- The CFD analyses performed as part of this study implement a simplistic boundary condition of a steady inflow velocity with a constant turbulence intensity. The inclusion of the ABL, along with models which account for gust modelling, and the airborne system’s reaction to such conditions must be investigated. Additionally, analyses must also include the effect of the performance of the turbine on various operational parameters like Reynolds number, turbulence intensity, and many more.
- Regarding aerostat sizing, given that it is in its initial design stage, details about the dynamics of the system are unavailable, which are necessary to size and design stabilization systems. The effect of various design parameters on the operational performance of the aerostat must be studied, along with developing a 6-DOF model to assess the complete dynamics of the aerostat and obtain critical data regarding its stability parameters. Additional studies on the material selection, envelope petal shaping, studies associated with confluence lines, ground station design and control must also be performed.
- The current study assumes that the payload is rigidly fixed with the aerostat, and any angular variation experienced by the aerostat is also experienced by the payload, and vice-versa. This is not entirely true in real life, and the rigid body dynamics of the complete airborne system must be assessed. Also, the current study assumes a payload mass based on an estimate from CAD software, does not include proper structural analyses and material analyses, and must be done in the future.
- The current study does not consider an rooftop setup from which the system is deployed, but rather provides a conceptual basis for such a system, which could be implemented in such environments.
- The current study does not highlight the safety measures needed to be employed. While the need for payload recovery devices and rapid deflation devices have been highlighted as necessary requirements for the rapid deflation of the aerostat and the recovery of the payload in case of emergencies, in-depth analysis on the safety requirement must be done so as to prepare for possible failure scenarios like heavy winds, lightning, tether failures and many more. Additionally, safety measures must be taken on ground as well so as to not cause harm to the urban populace in case of system failure. Safety systems must be equipped with redundancies to ensure proper working and a detailed analysis of the safety requirements and fail safes must be done before deploying the system.
- The current study is purely numerical and is in its conceptual stage and requires experimental studies to validate the results in the future.

Based on the results obtained from this study, along with accounting for the limitations of the current study, a structured framework is proposed for implementing aerostat-based WGD-CR-VAWT in the forthcoming section. This framework can be extended to implementing other augmentation devices and conventional VAWTs for airborne operation.

### Proposed framework for implementation of airborne VAWTs

The workflow in designing such systems from scratch is a twofold operation. Assuming that information regarding the power requirements, operational altitude, and deployment location are already known, two things are to be done simultaneously. One would be the sizing and sizing and design of the airborne system, whereas the other would be associated with the assessment of the area of deployment. The current workflow is a derivative of the proposed workflow in Ref.^100^.

The design of the CR-VAWT, based on the power requirements, can be done using the methodology prescribed in Ref.^28^ or using other optimization techniques to arrive at the final turbine design. Once the design of the turbine is finalized, studies regarding the impact of various operational parameters on the performance of the CR-VAWT must be performed. This must also include the assessment of the performance of CR-VAWTs under different inflow conditions. With all this known, the sizing of the WGD is done. Once again, any methodology and optimization technique can be implemented to arrive at the final design of the WGD, which can be used for power augmentation. Specific emphasis on optimizing the WGDs can also include improvement of CR-VAWT’s performance by suppressing or mitigating dynamic stall and various other parameters. The target function in the optimization process must be chosen accordingly for such scenarios. Once the WGD is designed, a similar set of analyses must be performed to assess the performance of the system and its reaction to different operational parameters. At this stage, a complete dataset of various global and local performance parameters and their correlation with the operational parameters must be available. Up to this stage, analyses are performed using computational methods to save time and resources. These computational results must be validated using experiments, and the experimental campaign can also help obtain data which may not have been observed in numerical simulations and can provide correction factors to the numerical solution to account for the variations. Studies associated with material selection and structural analysis can be performed at this stage to obtain various structural data associated with the system.

Once the mass of the WGD-CR-VAWT, which would act as the payload, is known, the aerostat can be sized. The current study implements the force-weight approach to design an oblate spheroid aerostat. Different aerostat envelope profiles can be chosen based on requirements, and appropriate methodology for its sizing can be implemented. Nonetheless, the basic process flow in the sizing of the aerostat would remain the same as discussed in this study, which would include an iterative procedure of size estimation and provide mass breakdown of individual components. With the sizing operations performed, shape optimization techniques can be implemented to further optimize the aerostat profile’s shape. Once the conceptual design of the aerostat profile is obtained, stability analyses are performed to obtain the sizing of the stabilization systems, which could be fins, kites or sails, which can provide stability to the system. At this stage, the dynamics of the aerostat are obtained to better understand the state of the system under the influence of winds. Appropriate material selection for the envelope, tether and other components is done at this stage, and the design is reiterated after accounting for these changes. Additional changes, like including recovery devices and sensors, must also be accounted for during the design process. Other analyses like tether profile analyses, net static lift variation with different design parameters, and aerostat motion in the presence of different inflow conditions must be performed at this stage. Finally, complete analyses accounting for the mutual interaction between the payload and the aerostat must be performed, and changes, if necessary, must be made. Any changes in the performance parameters of the CR-VAWT in the presence of the aerostat must also be accounted for at this stage.

The other part of the study, which can be performed in parallel, would be assessing the deployment area. The first and foremost step is the wind resource assessment, which includes data on windspeeds, directions, turbulence intensity, diurnal variations in atmospheric conditions and surface roughness. Deployment in an urban setup require additional studies to assess the effect of the urban canopy layer and the urban boundary layer on the available wind resources. The other would be rooftop area assessment. The implementation of these systems can be done from high rise buildings, which would reduce the overall weight of the system, due to the reduction in tether length, compared to deploying the system from ground level to the same altitude. The roofs of the buildings must have sufficient space to house the winch system and the ground station for the system. Additionally the rooftop station and the winch system should be connected to emergency backup power supply to ensure that the airborne system can be safely recovered in the case of system failures and pose minimal threat to the neighbourhood. Data associated with wind resources can be obtained from short-term or long-term measurements. The usage of both these data and localized analyses of the specified area is advised to get a better understanding of the wind resources with added accuracy. Few methods on wind resource assessment for rooftop wind turbines along with procedures are assessed and summarized Ref.^54^.

The remaining space on the rooftops can be used to implement other renewable energy technologies, one of them being photovoltaic cells, which would further improve the sustainable nature of the building and reduce its carbon footprint. An added advantage of implementing the wind turbines in an airborne configuration as theorized in this paper, would be the use of lesser number of turbines than the conventional method of implementing the same on the rooftops, occupying more space, requiring more turbines for the same net power output. This would reduce costs in terms of installation and maintenance. Another key factor would be possible the noise emission reduction, which is a key factor for urban areas. The presence of WGDs showed possible indicators for tonal noise reduction with the suppression of tip vortex shedding. The tonal noise reduction must be confirmed with detailed aeroacoustic analysis. Additionally, in the airborne configuration, the presence of high windspeeds and would enable the dissipation of the wake generated at a faster rate, reducing broadband noise emissions as well. Additionally, with the system implemented at high altitudes, flow interference from urban infrastructure would be minimal, allowing for a more cleaner incoming wind for efficient and consistent operation.

Once these two parts of the study are performed, data from both can be combined, and necessary calculations and testing can be performed. Until now, the inflow conditions have been assumed, and the CR-VAWTs and aerostats have been designed and analyzed. More accurate inflow conditions of the deployment area are used to obtain data on the performance of the airborne system, and changes in performance must be accordingly accounted for. Changes must be implemented to the system. Once all these data are obtained, manufacturing and field testing of these systems are proposed to get more accurate data. Once the field testing is complete, any fine-tuning of the system is performed, and the system is ready for deployment. The current framework is iterative, and changes and variations must be added and accounted for at the various stages of the design. The proposed framework does not explicitly discuss specific studies associated with vibrations, noise signature, power transmission systems, costs, connection to electrical grids and many more. These subsystems are accounted for in the later stages of the design and implementation process, specifically after testing the viability of the proposed system. Nevertheless, data associated with these subsystems must be included in the necessary stages of the system’s design and implementation. Deriving from the concept of implementation of aerostats for emergency communications during times of natural disasters, the same can be extended for emergency power supply, where the proposed system can be deployed for emergency power supply, and the portable nature of the system, along with the simplistic construction and installation makes it a viable candidate for the same. Finally communication devices could also be integrated with the current system to provide both emergency power and communication.

The above-mentioned framework is open to changes and adaptations based on requirements. It is not a complete framework and requires constant updates due to the developing field of airborne wind energy. Irrespective of the open nature of the framework, it provides a reasonable basis and methodology for the design, analysis and implementation of aerostat-based WGD-CR-VAWTs, which can be extended to the implementation of other augmentation devices and VAWTs with additional modifications based on the design.

## Conclusions and future work

The following study focuses on the design and analysis of a Wind Gathering device for a Contra-Rotating VAWT and an oblate spheroid aerostat, which can be used to carry the WGD-CR-VAWT to higher altitudes and to harness more power and act as an airborne wind energy system. The study included a CFD-Taguchi-based optimization of the WGD to provide power augmentation to the CR-VAWT and the conceptual design of an oblate spheroid aerostat using the force-weight approach required to carry said system to a specified altitude above ground level. The following inferences were made:

- Taguchi analysis of the WGD revealed that the semi-minor axis had the highest impact on the optimization and the overall power augmentation capability of the WGD, followed by the semi-major axis and the tip gap, respectively.
- CR-VAWT equipped with the optimal WGD provided an average of 32% more power than its non-ducted counterpart between *λ* = 2 − 4, with the highest augmentation of 34.5% at *λ* = 2*.*5.
- The presence of WGD helps blades experience higher instantaneous torque, especially in the upwind segment, with an average augmentation in torque of 40% in the said segment, leading to higher turbine efficiency.
- WGDs, while not beneficial in mitigating or suppressing dynamic stall, help augment the velocity across the rotor plane, due to which blades experience higher torque, thus providing higher power than their non-ducted counterparts while experiencing dynamic stall.
- WGDs are useful in mitigating blade tip losses due to the synergetic interaction between the blade tip vortices and the boundary layer of the WGD which act as a prime indicator for tonal noise reduction.
- From the force-weight approach for the design of the oblate spheroid aerostat to carry a 50 kg payload, the final size of the aerostat obtained from the iterative code has a volume of 92.1 m^3^, surface area of 115.67 m^2^, and a fineness ratio of 2.5, respectively.
- The above-mentioned aerostat provided a net static lift of 937.55 N, which accounts for 80% of the gross static lift generated. Additionally, the presence of excess tether was shown to be beneficial in countering the blow-by effects and maintaining the system at its operational altitude.
- Under the influence of a skewed flow, the airborne system, in the presence of a WGD, tends to produce higher power than its non-ducted counterpart. The airborne WGD-CR-VAWT showed reduced but efficient power production, even in highly skewed flows, by providing an average 17% augmentation beyond *α* = 5°and up to 58% more power at a highly skewed angle of 10°than its open counterpart at the same conditions.
- The net space occupied on the rooftop by the proposed system would be less compared to implementing wind turbines on rooftops in the conventional method, paving way for integrating other renewable energy technologies like photovoltaics, thus making the building more sustainable and dependent on green energy. Additionally, the portable nature of the system, and minimal effort required in installation makes it a viable candidate for a emergency power system in case of natural disasters.

This indicates that an airborne WGD-CR-VAWT has excellent potential as an airborne wind energy system, which can be implemented in various rural and urban setups to exploit the wind resources available and harness more power for human consumption. Given its potential, a framework for future studies on implementing airborne WGD-CR-VAWT has been provided. This framework can be extended to the implementation of different augmentation mechanisms and conventional VAWTs (“Proposed Framework for Implementation of Airborne VAWTs”). While the study explores the system’s viability, the limitations (provided in “Limitations”) of the study will be addressed in the future. Additional studies associated with vibrations, noise, structures, material selection, generator design, and mechanisms for power transmission to the ground station will also be addressed in future work.

## A mathematical calculations associated with aerostat sizing

The sizing of the aerostat begins with assuming a value for the semi-major axis and the fineness ratio (*φ*) of the oblate spheroid. From this, the value of the semi-minor axis (*b*) is calculated as

2 $$b = \frac{a}{\varphi }$$

From this, the angular eccentricity (*e*_*env*_), surface area (*S*_*env*_) and volume (*V*_*env*_) of the oblate spheroid is obtained from

3 $$e_{env} = 1 - \frac{{b^{2} }}{{a^{2} }}$$

4 $$S_{env} = 2\pi a^{2} + \frac{{\pi b^{2} }}{{e_{env} }}\log \frac{{(1 + e_{env} )}}{{1 - e_{env} }}$$

5 $$V_{env} = \frac{4}{3}\pi a^{{2}} b$$

With the values of ambient pressure and temperature obtained as inputs based on operational altitude above MSL, the saturated vapour pressure (*e*_*s*_) is calculated using the Arden-Buck equation and is given by

6 $${\text{e}}_{{\text{s}}} { = 611}{\text{.2 }}_{e}^{{\text{l}}} \left( {{18}{\text{.678 - }}\frac{{{\text{T - 273}}{.15}}}{{{234}{\text{.5}}}}} \right) \, \frac{{{\text{T - 273}}{.15}}}{{{257}{\text{.4 - (T - 273}}{.15)}}}$$

Based on the obtained value of *e*_*s*_, the actual vapour pressure, which is a function of the relative humidity, is calculated as

7 $$e = \frac{{{\text{RHe}}_{{\text{s}}} }}{100}$$

Using the Force-Weight approach as mentioned in Taylor, the total buoyant force generated, also called the gross static lift, *L*_*g*_, is given by

8 $$L_{g} = {1}.{15}KV_{env} \frac{{{\text{P}} - {\text{e}}({1} - RD_{wv} )}}{T}$$

where *RD*_*wv*_ represents the relative density of water vapour, and K represents the aerostatic constant with a value of 0.03416 K/m, respectively. Note that the constant 1.15 represents the 15% increment due to free lift.

With the values of inflation fraction, *I*, purity percentage, *Y* , relative density of LTA gas, *RD*_*lta*_ superpresure, *P*_*sh*_ and superheat, *T*_*sh*_, the weight of the lifting gas required to produce above said *L*_*g*_ can be found from

9 $$W_{lta} = IKV_{env} \frac{{{\text{P}} + P_{sh} }}{{T + T_{sh} }}({1} - Y({1} - RD_{lta} ))$$

The weight of the ballonet required for the aerostat is given as

10 $$W_{ba} = KV_{env} ({1} - I) \frac{{{\text{P}} + P_{sh} - e({1} - RD_{wv} )}}{{T + T_{sh} }}$$

The weight of the envelope material required for the oblate spheroid aerostat, accounting for 15% excess for seams and overlaps, is given by

11 $$W_{env} = {1}.{15}S_{env} \rho_{env} g \times 0.00{1}$$

The weight of the tether, accounting for a 30% excess tether to account for blow-by and drifting in the presence of ambient winds, is given by

12 $$W_{th} = {1}.{3}\rho_{th} H_{AGL} g \times 0.00{1}$$

Once all these values are obtained, the net static lift *L*_*net*_ is given by

13 $$L_{net} = L_{g} - W_{lta} - W_{ba}$$

With the value of *L*_*net*_, *W*_*th*_ and *W*_*env*_ available, the final payload weight available (*W*_*pay*_), accounting for 20% weight due to miscellaneous items (*W*_*misc*_) is given as

14 $$W_{rem} = L_{net} - W_{env} - W_{th}$$

15 $$W_{misc} = 0.{2}W_{rem}$$

16 $$W_{pay} = 0.{8}W_{rem}$$

The iterative code keeps running until the value of *W*_*pay*_ and the user input payload weight matches or at least meets the convergence criterion. Upon meeting said convergence conditions, the final value of the semi-major and semi-minor axis of the oblate spheroid is obtained as the output along with other geometrical outputs and mass of individual components. This data can then be used to design the aerostat and proceed forward with various other studies associated with aerostats and their implementation.

## Data Availability

The datasets generated during and/or analyzed during the current study are available from the first author (JR) on reasonable request.

## Abbreviations

*α*,AoA: Angle of attack (°)
*ε*: Augmentation ratio (*−*)
*λ* ,TSR: Tip speed ratio (*−*)
*µ*: Dynamic viscosity (*−*)
*ω*: Specific dissipation rate (1*/s*)
*C*_*P*_: Mean power coefficient (*−*)
*ρ*: Density (kg/m^3^)
*σ*: Solidity (*−*)
*θ*: Azimuth angle (°)
*Ω*: Rotational speed (rad/s)
*a*: Semi-major axis (m)
*A*_*g*_: Axial gap (cm)
*b*: Semi-minor axis (m)
*c*: Chord (m)
*C*_*m*_: Torque/moment coefficient (*−*)
*D*_*t*_: Turbine diameter (*m*)
*E*_*i*_: Extent of impact/weightage (*−*)
*H*_*b*_: Blade height (*m*)
*k*: Turbulent kinetic energy (m^2^/s^2^)
*N*_*b*_: Number of blades (*−*)
*Re*_*c*_: Chord-based Reynolds number (*−*)
*Re*_*D*__*t*_: Turbine diameter-based Reynolds number (*−*)
*rms*: Root mean square (*−*)
*S/N*: Signal-to-noise ratio (*−*)
*S/N*: Signal-to-noise (*−*)
*t*: Tip gap (*m*)
*U*_∞_: Freestream velocity (*m/s*)
AGL: Above ground level (*m*)
AR: Aspect ratio (*−*)
CFD: Computational fluid dynamics (*−*)
DSV: Dynamic stall vortex (*−*)
HAWT: Horizontal axis wind turbine (*−*)
LES: Large Eddy simulation (*−*)
LTA: Lighter than air (*−*)
MSL: Mean sea level (*m*)
OA: Orthogonal array (*−*)
RANS: Reynolds-averaged Navier–Stokes (*−*)
TI: Turbulence intensity (*−*)
VAWT: Vertical axis wind turbine (*−*)
WGD: Wind gathering device (−)

## References

[1] Tjahjana, D. D. D. P., Purbaningrum, P. & Hadi, S. Yoga arob wicaksono, and dimas adiputra. The study of the influence of the diameter ratio and blade number to the performance of the cross flow wind turbine by using 2d computational fluid dynamics modeling. In *AIP Conference Proceedings*, vol. 1931, 030034 (1931).

[2] Castelli, M. R., Englaro, A. & Benini, E. The darrieus wind turbine: Proposal for a new performance prediction model based on cfd. *Energy* **36**, 4919–4934 (2011).

[3] Galinos, C., Larsen, T. J., Madsen, H. A. & Paulsen, U. S. Vertical axis wind turbine design load cases investigation and comparison with horizontal axis wind turbine. *Energy Proc.* **94**, 319–328 (2016).

[4] Liu, J., Lin, H. & Zhang, J. Review on the technical perspectives and commercial viability of vertical axis wind turbines. *Ocean. Eng.* **182**, 608–626 (2019).

[5] Zhang, S., Du, B., Ge, M. & Zuo, Y. Study on the operation of small rooftop wind turbines and its effect on the wind environment in blocks. *Renew. Energy* **183**, 708–718 (2022).

[6] Lu, L. & Sun, K. Wind power evaluation and utilization over a reference high-rise building in urban area. *Energy Build.* **68**, 339–350 (2014).

[7] Tabrizi, A. B., Whale, J., Lyons, T. & Urmee, T. Performance and safety of rooftop wind turbines: Use of cfd to gain insight into inflow conditions. *Renew. Energy* **67**, 242–251 (2014).

[8] Liu, Q. et al. Effects of trailing-edge movable flap on aerodynamic performance and noise characteristics of vawt. *Energy* **189**, 116271 (2019).

[9] Hamada, K., Smith, T., Durrani, N., Qin, N. & Howell, R. Unsteady flow simulation and dynamic stall around vertical axis wind turbine blades. In *46th AIAA Aerospace Sciences Meeting and Exhibit*, 1319 (2008).

[10] Abu-Hamdeh, N. H. & Almitani, K. H. Construction and numerical analysis of a collapsible vertical axis wind turbine. *Energy Convers. Manag.* **151**, 400–413 (2017).

[11] Visbal, M. R. & Garmann, D. J. Analysis of dynamic stall on a pitching airfoil using high-fidelity large-eddy simulations. *AIAA J.* **56**, 46–63 (2018).

[12] Simão Ferreira, C., Van Kuik, G., Van Bussel, G. & Scarano, F. Visualization by piv of dynamic stall on a vertical axis wind turbine. *Exp. fluids* **46**, 97–108 (2009).

[13] Bianchini, A., Balduzzi, F., Ferrara, G. & Ferrari, L. Virtual incidence effect on rotating airfoils in darrieus wind turbines. *Energy Convers. Manag.* **111**, 329–338 (2016).

[14] Sridhar, S., Joseph, J. & Radhakrishnan, J. Implementation of tubercles on vertical axis wind turbines (vawts): An aerodynamic perspective. *Sustain. Energy Technol. Assess.* **52**, 102109 (2022).

[15] Mohamed, O. S., Melani, P. F., Balduzzi, F., Ferrara, G. & Bianchini, A. An insight on the physical mechanisms responsible of power augmentation in a pair of counter-rotating darrieus turbines. *Energy Convers. Manag.* **284**, 116991 (2023).

[16] Chen, Y. et al. Investigation of pitch angles on the aerodynamics of twin-vawt under staggered arrangement. *Ocean. Eng.* **254**, 111385 (2022).

[17] Lam, H. & Peng, H. Measurements of the wake characteristics of co-and counter-rotating twin h-rotor vertical axis wind turbines. *Energy* **131**, 13–26 (2017).

[18] Ahmadi-Baloutaki, M., Carriveau, R. & Ting, D. S. A wind tunnel study on the aerodynamic interaction of vertical axis wind turbines in array configurations. *Renew. Energy* **96**, 904–913 (2016).

[19] Sahebzadeh, S., Rezaeiha, A. & Montazeri, H. Vertical-axis wind-turbine farm design: Impact of rotor setting and relative arrangement on aerodynamic performance of double rotor arrays. *Energy Rep.* **8**, 5793–5819 (2022).

[20] Shaaban, S., Albatal, A. & Mohamed, M. Optimization of h-rotor darrieus turbines’ mutual interaction in staggered arrangements. *Renew. Energy* **125**, 87–99 (2018).

[21] Müller, S., Muhawenimana, V., Wilson, C. A. & Ouro, P. Experimental investigation of the wake characteristics behind twin vertical axis turbines. *Energy Convers. Manag.* **247**, 114768 (2021).

[22] Vergaerde, A. et al. Experimental characterisation of the wake behind paired vertical-axis wind turbines. *J. Wind. Eng. Ind. Aerodyn.* **206**, 104353 (2020).

[23] Dabiri, J. O. Potential order-of-magnitude enhancement of wind farm power density via counter-rotating vertical-axis wind turbine arrays. *J. Renew. Sustain. Energy* **3**, 043104 (2011).

[24] Poguluri, S. K., Lee, H. & Bae, Y. H. An investigation on the aerodynamic performance of a co-axial contra-rotating vertical-axis wind turbine. *Energy* **219**, 119547 (2021).

[25] Lee, H., Poguluri, S. K. & Bae, Y. H. Development and verification of a dynamic analysis model for floating offshore contra-rotating vertical-axis wind turbine. *Energy* **240**, 122492 (2022).

[26] Didane, D. H., Rosly, N., Zulkafli, M. F. & Shamsudin, S. S. Performance evaluation of a novel vertical axis wind turbine with coaxial contra-rotating concept. *Renew. Energy* **115**, 353–361 (2018).

[27] Didane, D. H., Rosly, N., Zulkafli, M. F. & Shamsudin, S. S. Numerical investigation of a novel contra-rotating vertical axis wind turbine. *Sustain. Energy Technol. Assess.* **31**, 43–53 (2019).

[28] Radhakrishnan, J., Sridhar, S., Zuber, M., Ng, E. Y. & Shenoy, S. Design optimization of a contra-rotating vawt: A comprehensive study using taguchi method and cfd. *Energy Convers. Manag.* **298**, 117766 (2023).

[29] Rezaeiha, A., Montazeri, H. & Blocken, B. Active flow control for power enhancement of vertical axis wind turbines: Leading-edge slot suction. *Energy* **189**, 116131 (2019).

[30] Post, M. L. & Corke, T. C. Separation control using plasma actuators: Dynamic stall vortex control on oscillating airfoil. *AIAA J.* **44**, 3125–3135 (2006).

[31] Shun, S. & Ahmed, N. Wind turbine performance improvements using active flow control techniques. *Proc. Eng.* **49**, 83–91 (2012).

[32] Greenblatt, D., Schulman, M. & Ben-Harav, A. Vertical axis wind turbine performance enhancement using plasma actuators. *Renew. Energy* **37**, 345–354 (2012).

[33] Greenblatt, D. & Lautman, R. Inboard/outboard plasma actuation on a vertical-axis wind turbine. *Renew. Energy* **83**, 1147–1156 (2015).

[34] Wang, Z. & Zhuang, M. Leading-edge serrations for performance improvement on a vertical-axis wind turbine at low tip-speed-ratios. *Appl. Energy* **208**, 1184–1197 (2017).

[35] Wang, Z., Wang, Y. & Zhuang, M. Improvement of the aerodynamic performance of vertical axis wind turbines with leading-edge serrations and helical blades using cfd and taguchi method. *Energy Convers. Manag.* **177**, 107–121 (2018).

[36] Zhu, H., Hao, W., Li, C. & Ding, Q. Numerical study of effect of solidity on vertical axis wind turbine with gurney flap. *J. Wind. Eng. Ind. Aerodyn.* **186**, 17–31 (2019).

[37] Hansen, M. O. L. et al. Aerodynamically shaped vortex generators. *Wind Energy* **19**, 563–567 (2016).

[38] Zhao, Z. et al. Researches on vortex generators applied to wind turbines: A review. *Ocean. Eng.* **253**, 111266 (2022).

[39] Bianchini, A., Balduzzi, F., Di Rosa, D. & Ferrara, G. On the use of gurney flaps for the aerodynamic performance augmentation of darrieus wind turbines. *Energy Convers. Manag.* **184**, 402–415 (2019).

[40] Sasson, B. & Greenblatt, D. Effect of leading-edge slot blowing on a vertical axis wind turbine. *AIAA J.* **49**, 1932–1942 (2011).

[41] Heine, B., Mulleners, K., Joubert, G. & Raffel, M. Dynamic stall control by passive disturbance generators. *AIAA J.* **51**, 2086–2097 (2013).

[42] Joseph, J. & Sathyabhama, A. Leading edge tubercle on wind turbine blade to mitigate problems of stall, hysteresis, and laminar separation bubble. *Energy Convers. Manag.* **255**, 115337 (2022).

[43] Zhang, T.-T. et al. Winglet design for vertical axis wind turbines based on a design of experiment and cfd approach. *Energy Convers. Manag.* **195**, 712–726 (2019).

[44] Li, Y. et al. Aerodynamic characteristics of straight-bladed vertical axis wind turbine with a curved-outline wind gathering device. *Energy Convers. Manag.* **203**, 112249 (2020).

[45] Li, Y., Tong, G., Zhao, B., Feng, F. & Tagawa, K. Study on aerodynamic performance of a straight-bladed vawt using a wind-gathering device with polyline hexagonal pyramid shape. *Front. Energy Res.* **10**, 790777 (2022).

[46] Nobile, R., Vahdati, M., Barlow, J. F. & Mewburn-Crook, A. Unsteady flow simulation of a vertical axis augmented wind turbine: A two-dimensional study. *J. Wind. Eng. Ind. Aerodyn.* **125**, 168–179 (2014).

[47] Chong, W. et al. The design, simulation and testing of an urban vertical axis wind turbine with the omni-direction-guide-vane. *Appl. Energy* **112**, 601–609 (2013).

[48] Wang, X. et al. Preliminary performance tests and simulation of a v-shape roof guide vane mounted on an eco-roof system. *Energies* **11**, 2846 (2018).

[49] Hashem, I. & Mohamed, M. Aerodynamic performance enhancements of h-rotor darrieus wind turbine. *Energy* **142**, 531–545 (2018).

[50] Dessoky, A., Bangga, G., Lutz, T. & Krämer, E. Aerodynamic and aeroacoustic performance assessment of h-rotor darrieus vawt equipped with wind-lens technology. *Energy* **175**, 76–97 (2019).

[51] Ranjbar, M. H. et al. Power enhancement of a vertical axis wind turbine equipped with an improved duct. *Energies* **14**, 5780 (2021).

[52] Stull, R. B. *An Introduction to Boundary Layer Meteorology*, vol. 13 (Springer Science & Business Media, 1988).

[53] Monin, A. The atmospheric boundary layer. *Annu. Rev. Fluid Mech.* **2**, 225–250 (1970).

[54] Vadhyar, A., Sridhar, S., Reshma, T. & Radhakrishnan, J. A critical assessment of the factors associated with the implementation of rooftop vawts: A review. *Energy Convers. Manag. X* 100563 (2024).

[55] Radünz, W. C., Mattuella, J. M. L. & Petry, A. P. Wind resource mapping and energy estimation in complex terrain: A framework based on field observations and computational fluid dynamics. *Renew. Energy* **152**, 494–515 (2020).

[56] Cherubini, A., Papini, A., Vertechy, R. & Fontana, M. Airborne wind energy systems: A review of the technologies. *Renew. Sustain. Energy Rev.* **51**, 1461–1476 (2015).

[57] Legaignoux, D. M. & Legaignoux, B. T. Propulsive wing with inflatable armature (1987). US Patent 4,708,078.

[58] Zhang, J., Zou, N. & Zhou, W.-L. System and method for umbrella power generation (2013). US Patent 8,405,244.

[59] Perkovic´, L., Silva, P., Ban, M., Kranjcˇevic´, N. & Duic´, N. Harvesting high altitude wind energy for power production: The concept based on magnus’ effect. *Appl. energy* **101**, 151–160 (2013).

[60] Dunker, S. Ram-air wing design considerations for airborne wind energy. In *Airborne Wind Energy*, 517–546 (Springer, 2013).

[61] Ippolito, M. Aeolian system for converting energy through power wing airfoils. *Eur. Pat. Appl. EP2463516A1* (2013).

[62] Milanese, M., Fagiano, L. & Gerlero, I. Actuating systems for controlling the flight of a power wing profile for conversion of wind energy into electrical or mechanical energy (2016). US Patent 9,366,225.

[63] Kruijff, M. & Ruiterkamp, R. A roadmap towards airborne wind energy in the utility sector. *Airborne Wind. Energy: Adv. Technol. Dev. Res.* 643–662 (2018).

[64] Loyd, M. L. Crosswind kite power (for large-scale wind power production). *J. Energy* **4**, 106–111 (1980).

[65] Loyd, M. L. Wind driven apparatus for power generation (1981). US Patent 4,251,040.

[66] Bevirt, J. Tethered airborne power generation system with vertical take-off and landing capability (2010). US Patent App. 12/784,328.

[67] Glass, B. Power-augmenting shroud for energy-producing turbines (2012). US Patent 8,253,265.

[68] Upadhyay, P. Lighter than air wind turbine platform. In *AIAA Aviation 2019 Forum*, 2980 (2019).

[69] Roberts, B. W. Control system for a windmill kite (2011). US Patent App. 12/936,786.

[70] Nambiar, R. R., Dixit, M. & Pant, R. S. Methodology for conceptual sizing of a turbine aerostat for electrical power generation. In *AIAA AVIATION 2023 Forum*, 3792 (2023).

[71] Pant, R. *et al.* Design-build-fly of osirca: Oblate spheroid indoor remotely controlled airship. In *11th AIAA Aviation Technology, Integration, and Operations (ATIO) Conference, Including the AIAA Balloon Systems Conference and 19th AIAA Lighter-Than*, 6914 (2011).

[72] Gawande, V., Bilaye, P., Gawale, A., Pant, R. & Desai, U. Design and fabrication of an aerostat for wireless communication in remote areas. In *7th AIAA ATIO Conf, 2nd CEIAT Int’l Conf on Innov and Integr in Aero Sciences, 17th LTA Systems Tech Conf; followed by 2nd TEOS Forum*, 7832 (2007).

[73] Pant, R., Komerath, N. & Kar, A. Application of lighter-than-air platforms for power beaming, generation and communications. In *2011 International Symposium on Electronic System Design*, 242–247 (IEEE, 2011).

[74] Chauhan, T. H., Agarwal, S., Purohit, S. & Kumar, A. Wireless communications from high altitude platforms. *Int. J. Emerg. Technol. Adv. Eng.* **3**, 220–223 (2013).

[75] Sharma, N., Sehgal, R., Pant, R. S. & Sehgal, R. Design fabrication and deployment of a tethered aerostat system for aerial surveillance. In *National Level Conference on Advances in Aerial/Road Vehicle and its Application, MIT, Manipal*, 18–19 (2014).

[76] Sharma, V., Dusane, C. R., Verma, R. & Pant, R. S. Design, fabrication and testing of an aerostat system for last mile communication. In *AIAA Aviation 2019 Forum*, 2979 (2019).

[77] Dusane, C. R., Wani, A. V., Pant, R. S., Chakraborty, D. & Chakravarthy, B. An elevated balloon-kite hybrid platform for surveillance. In *23rd AIAA Lighter-Than-Air Systems Technology Conference*, 3995 (2017).

[78] Rezaeiha, A., Kalkman, I. & Blocken, B. Cfd simulation of a vertical axis wind turbine operating at a moderate tip speed ratio: guidelines for minimum domain size and azimuthal increment. *Renew. energy* **107**, 373–385 (2017).

[79] Rezaeiha, A., Montazeri, H. & Blocken, B. Towards optimal aerodynamic design of vertical axis wind turbines: Impact of solidity and number of blades. *Energy* **165**, 1129–1148 (2018).

[80] McNaughton, J. et al. A simple sliding-mesh interface procedure and its application to the cfd simulation of a tidal-stream turbine. *Int. J. Numer. Methods Fluids* **74**, 250–269 (2014).

[81] Hadžic´, H. *Development and application of finite volume method for the computation of flows around moving bodies on unstructured, overlapping grids*. Ph.D. thesis, Zugl.: Hamburg, Techn. Univ., Diss., 2005 (2006).

[82] Chesshire, G. & Henshaw, W. D. Composite overlapping meshes for the solution of partial differential equations. *J. Comput. Phys.* **90**, 1–64 (1990).

[83] Gemayel, D., Abdelwahab, M., Ghazal, T. & Aboshosha, H. Modelling of vertical axis wind turbine using large eddy simulations. *Results Eng.* 101226 (2023).

[84] Li, Y., Wang, W. & Okaze, T. Evaluation of polyhedral mesh performance for large-eddy simulations of flow around an isolated building within an unstable boundary layer. *Build. Environ.* **235**, 110207 (2023).

[85] Menter, F. R. Two-equation eddy-viscosity turbulence models for engineering applications. *AIAA J.* **32**, 1598–1605 (1994).

[86] Rezaeiha, A., Montazeri, H. & Blocken, B. On the accuracy of turbulence models for cfd simulations of vertical axis wind turbines. *Energy* **180**, 838–857 (2019).

[87] Kato, K. The modeling of turbulent flow around stationary and vibrating square cylinders. In *Proc. of 9th Symp. Turbulent Shear Flows*, 1041–1046 (1993).

[88] Blocken, B., van Druenen, T., Toparlar, Y. & Andrianne, T. Aerodynamic analysis of different cyclist hill descent positions. *J. Wind. Eng. Ind. Aerodyn.* **181**, 27–45 (2018).

[89] Menter, F. R. et al. A correlation-based transition model using local variables—part i: model formulation. *J. Turboma- chinery* **128**, 413–422 (2006).

[90] Menter, F., Langtry, R. & Völker, S. Transition modelling for general purpose cfd codes. *Flow Turbul. Combust.* **77**, 277–303 (2006).

[91] Blocken, B. et al. Aerodynamic drag in cycling pelotons: New insights by cfd simulation and wind tunnel testing. *J. Wind. Eng. Ind. Aerodyn.* **179**, 319–337 (2018).

[92] Langtry, R. B. *et al.* A Correlation-based transition model using local variables—Part II: test cases and industrial applications. *J. Turbomach.* 423–434 (2004).

[93] Taylor, J. A. Principles of aerostatics. *The Theory LTA Flight* (2014).

[94] Bagare, S. V., Joshi, A. & Pant, R. S. A methodology for sizing of a mini-aerostat system. In *AIAA AVIATION 2021 FORUM*, 2986 (2021).

[95] Krausman, J. Investigation of various parameters affecting altitude performance of tethered aerostats. In *11th Lighter-than-Air Systems Technology Conference*, 1625 (1995).

[96] Sharma, N., Mukhopadhyay, A., Sharma, V., Milind, M. & Pant, R. S. Design and field trials of a payload recovery device for tethered aerostats. In *Innovative Design, Analysis and Development Practices in Aerospace and Automotive Engineering: I-DAD 2014, February 22–24, 2014*, 79–84 (Springer, 2014).

[97] Vivek, P., Sharma, V. & Pant, R. An emergency deflation device for remotely controlled airships. *J. Airsh. Assoc.* 11–16 (2014).

[98] Bilaye, P., Gawande, V., Desai, U., Raina, A. & Pant, R. Low cost wireless internet access for rural areas using tethered aerostats. In *2008 IEEE Region 10 and the Third international Conference on Industrial and Information Systems*, 1–5 (IEEE, 2008).

[99] Wright, J. B. *Computer programs for tethered-Balloon System Design and Performance Evaluation*, vol. 76 (Air Force Geophysics Laboratory, Air Force Systems Command, United States, 1976).

[100] Radhakrishnan, J., Baloor, S. S., Zuber, M. & Sridhar, S. Aerial vertical axis wind turbine system and method thereof (2024). Indian Patent Office App. 202441105125.

